# 2-Phenyl-1*H*-pyrrole-3-carboxamide as a New Scaffold for Developing 5-HT_6_ Receptor Inverse Agonists with Cognition-Enhancing Activity

**DOI:** 10.1021/acschemneuro.1c00061

**Published:** 2021-03-11

**Authors:** Marcin Drop, Vittorio Canale, Séverine Chaumont-Dubel, Rafał Kurczab, Grzegorz Satała, Xavier Bantreil, Maria Walczak, Paulina Koczurkiewicz-Adamczyk, Gniewomir Latacz, Anna Gwizdak, Martyna Krawczyk, Joanna Gołębiowska, Katarzyna Grychowska, Andrzej J. Bojarski, Agnieszka Nikiforuk, Gilles Subra, Jean Martinez, Maciej Pawłowski, Piotr Popik, Philippe Marin, Frédéric Lamaty, Paweł Zajdel

**Affiliations:** † Faculty of Pharmacy, Jagiellonian University Medical College, 9 Medyczna Str., 30-688 Kraków, Poland; ‡ IBMM, Université de Montpellier, CNRS, ENSCM, 34095 Montpellier, France; § Institut de Génomique Fonctionelle, Université de Montpellier, CNRS, INSERM, 34094 Montpellier, France; ∥ Maj Institute of Pharmacology, Polish Academy of Sciences, 12 Smętna Str., 31-343 Kraków, Poland

**Keywords:** Cognition, 5-HT_6_ receptor, constitutive activity, inverse agonism, Cdk5 signaling, 2-phenyl-1*H*-pyrrole-3-carboxamide, novel object recognition test, attentional set shifting task

## Abstract

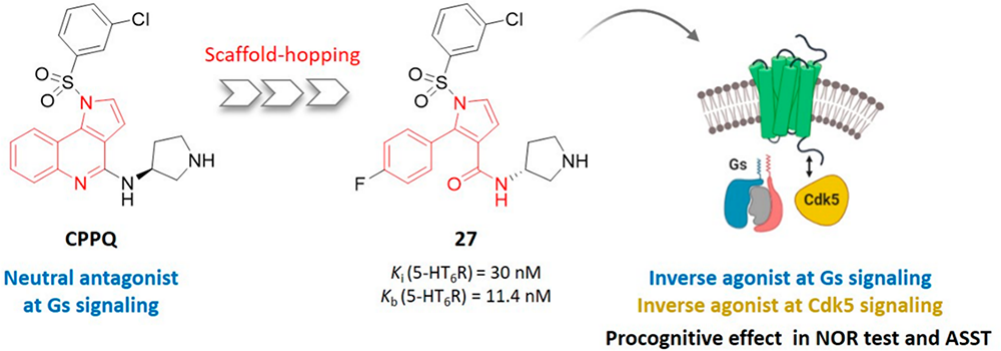

Serotonin type 6 receptor (5-HT_6_R) has gained particular interest as a promising target for treating cognitive deficits, given the positive effects of its antagonists in a wide range of memory impairment paradigms. Herein, we report on degradation of the 1*H*-pyrrolo[3,2-*c*]quinoline scaffold to provide the 2-phenyl-1*H*-pyrrole-3-carboxamide, which is devoid of canonical indole-like skeleton and retains recognition of 5-HT_6_R. This modification has changed the compound’s activity at 5-HT_6_R-operated signaling pathways from neutral antagonism to inverse agonism. The study identified compound **27** that behaves as an inverse agonist of the 5-HT_6_R at the Gs and Cdk5 signaling pathways. Compound **27** showed high selectivity and metabolic stability and was brain penetrant. Finally, **27** reversed scopolamine-induced memory decline in the novel object recognition test and exhibited procognitive properties in the attentional set-shifting task in rats. In light of these findings, **27** might be considered for further evaluation as a new cognition-enhancing agent, while 2-phenyl-1*H*-pyrrole-3-carboxamide might be used as a template for designing 5-HT_6_R inverse agonists.

## 1 Introduction

Cognitive decline and mental retardation are associated with several neurological and psychiatric disorders such as Alzheimer’s disease, Parkinson’s disease, schizophrenia, depression, Down syndrome, and autism spectrum disorders.^1,2^ The complexity and progressive nature of these diseases and the limited efficacy of the currently used drugs indicate the paramount need to develop novel therapeutic approaches. Among the different molecular targets, the 5-HT_6_ receptor (5-HT_6_R) has emerged as a particularly promising target to alleviate cognitive impairments.^3,4^

The 5-HT_6_R belongs to the family of G-protein-coupled receptors (GPCRs), which are canonically coupled to the adenylyl cyclase pathway.^5^ Recent studies have identified 125 candidate receptor partners, making the 5-HT_6_R one of the GPCRs with the most extensively characterized interactome.^6,7^ In addition to the canonical Gs-adenylyl cyclase signaling pathway, the 5-HT_6_R has been linked to cellular signaling cascades involved in cognitive processes and neurogenesis, such as mammalian target of rapamycin (mTOR) and cyclin-dependent kinase 5 (Cdk5) pathways. Indeed, enhanced mTOR activity under the control of the 5-HT_6_R contributes to cognitive deficits associated with schizophrenia^8^ and cannabis abuse during adolescence.^9^ Activation of Cdk5 signaling by the 5-HT_6_R plays a crucial role in the migration of cortical neurons and the initiation of neurite growth.^10^

Another important feature of the 5-HT_6_R is represented by a high level of constitutive activity. This particular property corresponds to the ability of the receptor to be spontaneously active in the absence of an agonist.^11^ The 5-HT_6_R constitutive activity was established for recombinant receptors expressed in cell lines^12^ and subsequently confirmed for native receptors in primary cultured neurons and mouse brain.^13^

The highest expression of 5-HT_6_Rs is found in the central nervous system regions involved in mnemonic functions such as the hippocampus, striatum, nucleus accumbens, and prefrontal cortex. Further lines of evidence demonstrated the expression of the 5-HT_6_R in the primary cilium, highlighting the involvement of the 5-HT_6_R in neuronal morphology.^14,15^ The cognitive-enhancing properties result from the blockade of 5-HT_6_Rs located on GABAergic neurons and promotion of corticolimbic release of acetylcholine and glutamate.^16^

**Figure 1:**
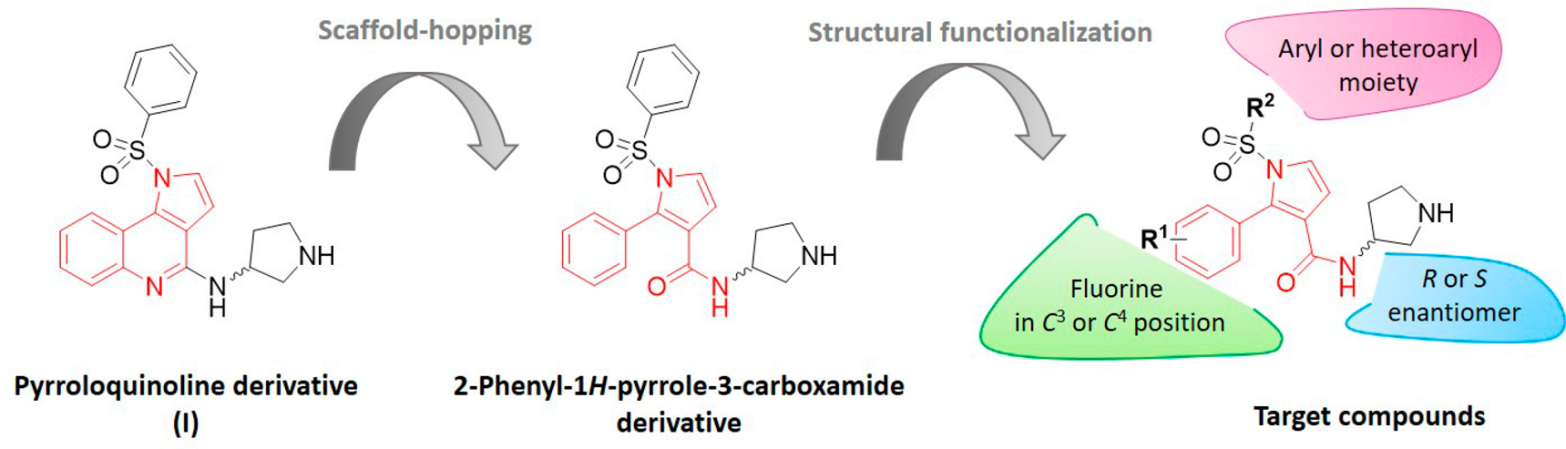
Replacement of 1*H*-pyrrolo[3,2-*c*]quinoline scaffold with 2-phenyl-1*H*-pyrrole-3-carboxamide and structural functionalization providing the target compounds.

Our recent interest in 5-HT_6_R ligands has culminated in the identification of (*S*)-1-[(3-chlorophenyl)sulfonyl]-4-(pyrrolidine-3-yl-amino)-1*H*-pyrrolo[3,2-*c*]quinoline (CPPQ), a pyrroloquinoline-derived potent and selective 5-HT_6_R neutral antagonist.^17,18^ Inspired by the activity of CPPQ,^9,13^ we applied the scaffold-hopping approach to replace the planar 1*H*-pyrrolo[3,2-*c*]quinoline skeleton with a more flexible 2-phenyl-1*H*-pyrrole-3-carboxamide in order to investigate the effect of a central core retraction on 5-HT_6_R affinity and functional activity. The chemical diversity around the new framework involved (*i*) introduction of a fluorine atom at the 2-phenyl fragment (R^1^), (*ii*) functionalization of *N*^1^ pyrrole with aryl- or heteroarylsulfonyl moieties (R^2^), and (*iii*) changing configuration at the 3-aminopyrrolidinyl moiety at the 3-carboxamide fragment (Figure 1).

The present manuscript reports on the identification of a promising arylsulfonamide of 2-phenylpyrrole-3-carboxamide analogue (**27**) which displays inverse agonist properties at 5-HT_6_R-operated Gs and Cdk5 signaling pathways. We also examined whether **27** is distributed to the brain and could reverse cognitive impairments in the novel object recognition (NOR) test under scopolamine-induced memory decline conditions and whether it could facilitate cognitive flexibility in the attentional set-shifting task (ASST) in rats.

## 2 Chemistry

**Scheme 1:**
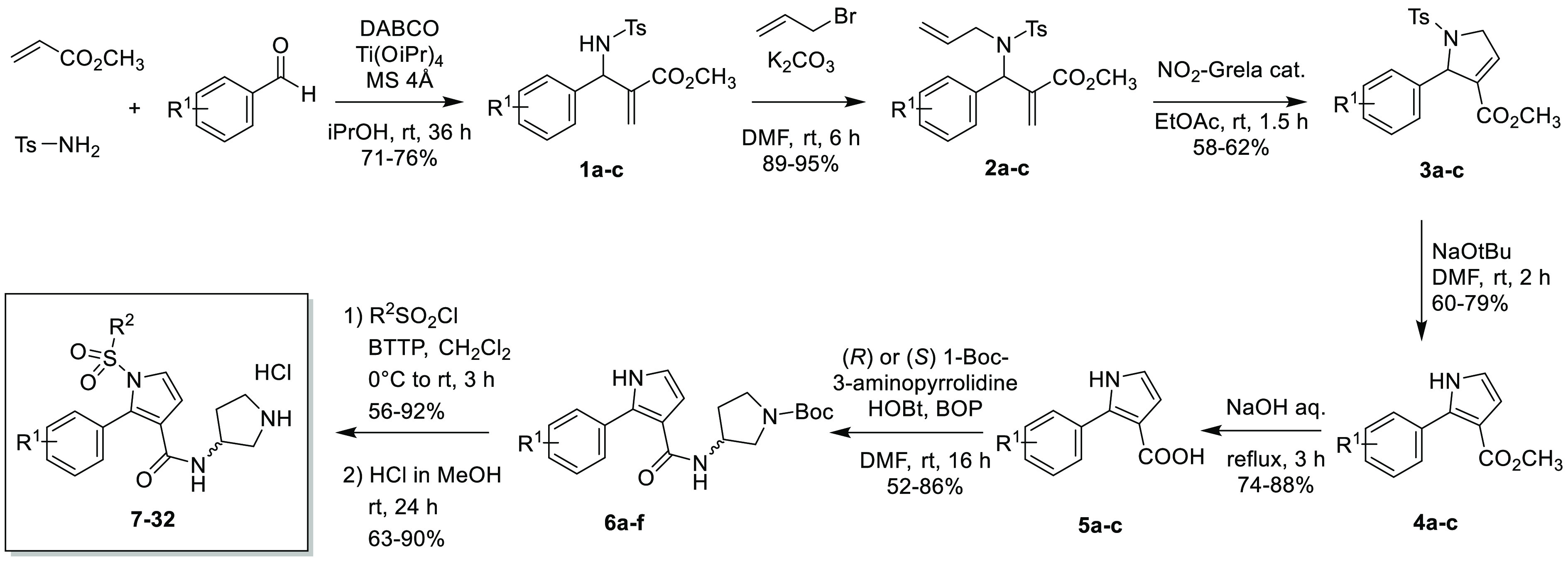
General Synthetic Route for the Preparation of Final Compounds **7**–**32**

The general synthetic route used to prepare final compounds **7**–**32** is summarized in Scheme 1. By use of our previously optimized procedures,^19,20^ intermediates **1**–**4** were synthesized via a four-step sequence involving (*i*) aza-Baylis–Hillman reaction, (*ii*) N-allylation, (*iii*) ring-closing metathesis, and (*iv*) removal of the tosyl group with simultaneous aromatization. Next, saponification of the esters **4a**–**c** in a refluxing aqueous solution of NaOH furnished the carboxylic acids **5a**–**c**. Amide coupling with (*R*) or (*S*) 1-Boc-3-aminopyrrolidine was then performed using 1-hydroxybenzotriazole (HOBt) and benzotriazole-1-yl-oxy-tris(dimethylamino)phosphonium hexafluorophosphate (BOP) as a carboxyl group activating agents. The obtained 2-aryl-1*H*-pyrrole-3-carboxamides **6a**–**f** were subsequently reacted with various arylsulfonyl chlorides in the presence of a phosphazene base P_1_-*t*-Bu-tris(tetramethylene) (BTPP). Finally, Boc-deprotection using methanolic solution of HCl resulted in target compounds **7**–**32** as corresponding hydrochloride salts.

## 3 Results and Discussion

### 3.1 Structure–Activity Relationship (SAR) Studies

To reveal common pharmacophore features for 5-HT_6_R antagonists,^21^ 2-phenyl-1*H*-pyrrole-3-carboxamide was used as an aromatic ring hydrophobic site instead of the previously used 1*H*-pyrrolo[3,2-*c*]quinoline scaffold. The applied new framework opened the possibility of introducing the second hydrophobic site linked to the central core by a sulfonyl group at the *N*^1^ position of pyrrole as a hydrogen bond acceptor and an alicyclic amine in the 3-carboxamide fragment providing a positively ionizable atom.

**Figure 2:**
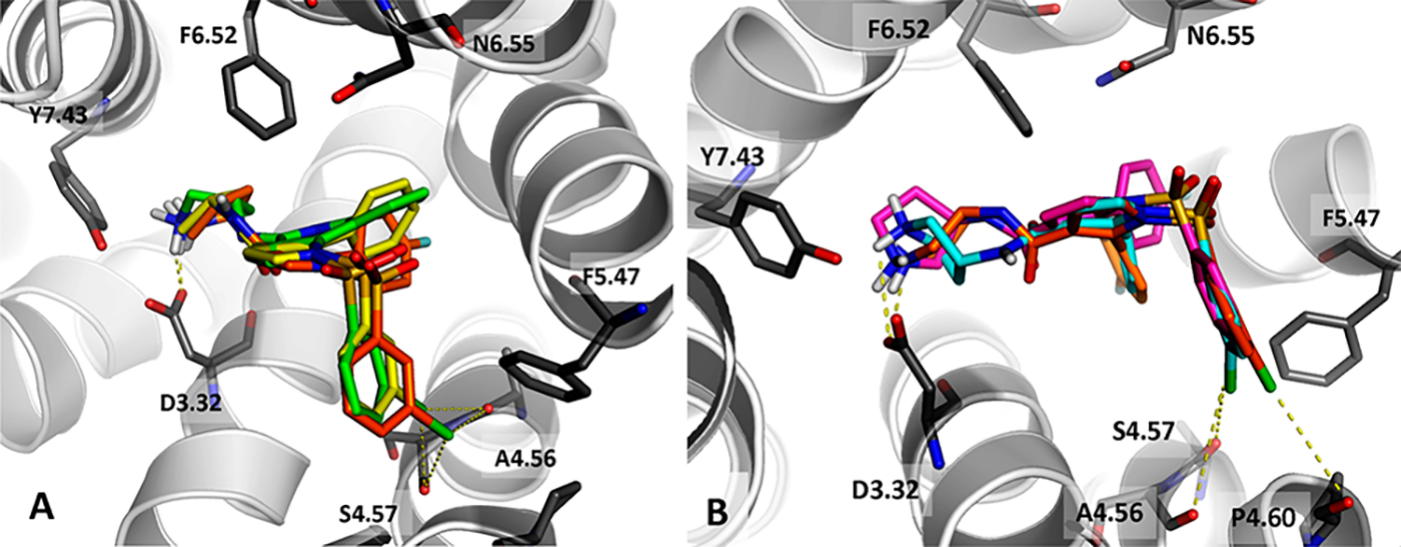
(A) Comparison of the binding mode of **18** (yellow) with **28** (orange) and CPPQ (green). (B) Comparison of the binding mode of **24** (magenta), **27** (cyan), and **28** (orange).

Final compounds were evaluated in the [^3^H]-LSD binding assay using HEK293 cells with stable expression of human 5-HT_6_R. The SAR studies initially revealed that the degradation of the 1*H*-pyrrolo[3,2-*c*]quinoline central core to 2-phenyl-1*H*-pyrrole-3-carboxamide significantly decreased the affinity of new derivatives **7** and **8** for 5-HT_6_R (**I**, *K*_i_ = 10 nM vs **7**, *K*_i_ = 208 nM; **8**, *K*_i_ = 106 nM) (Figure 1). Because the fluorine atom might affect the affinity at target protein as well as physicochemical and pharmacokinetic properties of compound,^22^ fluorine substitution at the 2-phenyl ring was applied. The introduction of fluorine in the *C*^4^ position (R^1^) maintained (**17** vs **27**; **18** vs **28**) or slightly increased (**7** vs **24**; **12** vs **26**) the affinity for 5-HT_6_R, while the *C*^3^ position was less preferable (**21** vs **17** and **27**). The analysis of the binding mode of **18** and **28** (Figure 2A) confirmed that the 2-phenyl moiety is placed in the hydrophobic cavity between transmembrane domains (TMs) 4–6.

Considering our previously reported data,^17,23,24^ which indicate that structural functionalization of the arylsulfonamide fragment (R^2^) highly impacts the affinity for the 5-HT_6_R, diverse substitution, i.e., halogen atom, alkyl, or alkoxyl moieties, was investigated. Generally, substitution in the *C*^3^ position at the phenylsulfonyl ring was beneficial for interaction with the 5-HT_6_R. Introduction of electron-withdrawing substituents such as fluorine, chlorine, trifluoromethyl, or the bulky trifluoromethoxy group significantly increased the affinity for 5-HT_6_R (**24** vs **26**; **8** vs **18**; **8** vs **14**; **8** vs **16**; respectively). Among electron-withdrawing substituents, chlorine was the most privileged one, increasing the affinity for the 5-HT_6_R up to 6-fold (**7** vs **17**; **8** vs **18**). This may result from the fact that halogen atoms stabilize the ligand–receptor complex by forming additional interactions and thus improve the affinity for 5-HT_6_Rs.^25^ Indeed, the analysis of the binding modes (Figure 2B) revealed that chlorine atom in *C*^3^ position at the phenylsulfonyl ring may form halogen bonding contacts with the carbonyl oxygen of A4.56 and S4.57, which were not detected for unsubstituted derivatives (**27** vs **24**). Interestingly, these amino acids were also indicated as halogen bonding hot spots in other classes of 5-HT_6_R ligands.^26,27^ On the other hand, the introduction of electron-donating substituents was less favorable for interaction with the 5-HT_6_R (**24** vs **25**), while a small methyl substituent was tolerated (**11**, *K*_i_ = 96 nM). The shifting of a chlorine atom or a methyl group from the *C*^3^ to *C*^2^ position decreased the affinity for the 5-HT_6_R (**10** vs **17**; **9** vs **11**). Surprisingly, a loss of affinity for the 5-HT_6_R was observed when the fluorine atom was moved from the *C*^3^ to *C*^4^ position (**26** vs **29**). Finally, the replacement of the phenyl fragment with five-membered heterocyclic moieties, namely, thien-2-yl (**19**) and 1-methyl-1*H*-pyrazol-4-yl (**20**) or bicyclic naphth-1-yl (**31**) and quinol-8-yl (**32**), was not suitable for interaction with the 5-HT_6_R (Table 1).

**Table 1:**
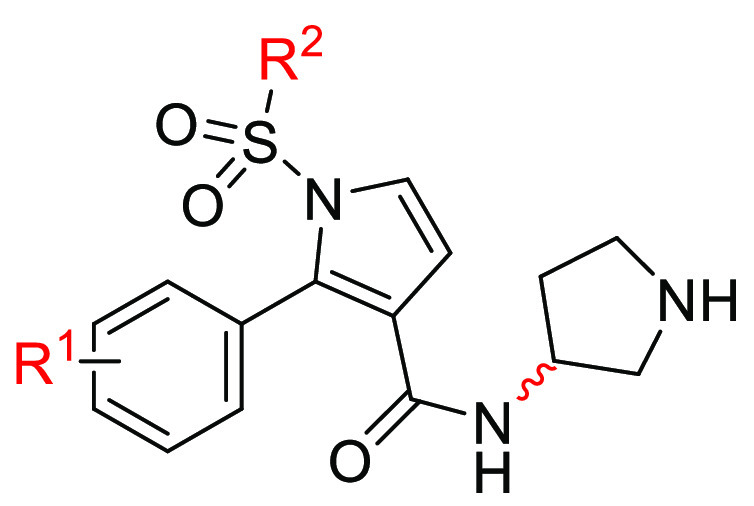
Binding Data of Synthesized Compounds **7**–**32** and Reference for the 5-HT_6_R

compd	R^1^	R^2^	*R*/*S*	*K*_i_ [nM],^a^ 5-HT_**6**_R
**7**	H	Ph	*R*	208
**8**	H	Ph	*S*	106
**9**	H	2-CH_3_-Ph	*R*	324
**10**	H	2-Cl-Ph	*R*	179
**11**	H	3-CH_3_-Ph	*R*	96
**12**	H	3-F-Ph	*R*	88
**13**	H	3-CF_3_-Ph	*R*	159
**14**	H	3-CF_3_-Ph	*S*	38
**15**	H	3-OCF_3_-Ph	*R*	52
**16**	H	3-OCF_3_-Ph	*S*	31
**17**	H	3-Cl-Ph	*R*	35
**18**	H	3-Cl-Ph	*S*	25
**19**	H	thien-2-yl	*S*	162
**20**	H	1-methyl-1*H*-pyrazol-4-yl	*S*	1463
**21**	3-F	3-Cl-Ph	*R*	98
**22**	3-F	3-Cl-Ph	*S*	28
**23**	3-F	4-F-Ph	*R*	853
**24**	4-F	Ph	*R*	102
**25**	4-F	3-OCH_3_-Ph	*R*	350
**26**	4-F	3-F-Ph	*R*	56
**27**	4-F	3-Cl-Ph	*R*	30
**28**	4-F	3-Cl-Ph	*S*	22
**29**	4-F	4-F-Ph	*R*	2577
**30**	4-F	4-F-Ph	*S*	638
**31**	4-F	naphth-1-yl	*S*	391
**32**	4-F	quinol-8-yl	*S*	1210
**I**^b^				10
CPPQ^b^				3

a Mean *K*_i_ values (SEM ± 19%) based on three independent binding experiments.

b Data taken from ref (17), where **I** is encoded as 9, and CPPQ is encoded as 14.

Because the stereochemical properties of compounds might modify the affinity for the 5-HT_6_R, both enantiomers of the pyrrolidin-3-yl fragment were investigated. With regard to their binding with target protein, a preference for the *S* enantiomers over its *R* counterparts was observed (Table 1). A comparison of the binding mode of **27** and **28** (Figure 2B) demonstrated modest differences in the orientation of the basic group, while both enantiomers created a strong salt bridge with D3.32.

Molecular docking was also used to study the binding mode of newly synthesized 2-phenyl-1*H*-pyrrole-3-carboxamides using CPPQ as a reference compound.^17^ Generally, the binding mode was coherent and showed a similar interaction pattern to CPPQ; i.e., the protonated fragment containing basic nitrogen formed a salt bridge with D3.32, the aromatic scaffold created CH−π interaction with F6.52/F6.51, and the terminal substituted phenyl ring expanded into a hydrophobic cavity formed by TMs 3, 4, and 5 and the extracellular loop 2 (ECL2). Comparison of the binding modes of **18** and **28** with CPPQ (Figure 2A) indicates that the phenyl moiety of the central core is twisted compared to the pyrroloquinoline system of CPPQ so that interaction with the aromatic cluster F6.52/F6.51 is further stabilized.

### 3.2 Selectivity Profiles of Selected Compounds

The most potent compounds (*K*_i_ ≤ 30 nM) exhibiting structural (**18**, **22**, **28**) and enantiomeric (**27**, **28**) diversity were further evaluated for their selectivity over serotonin receptors (5-HT_1A_R, 5-HT_2A_R, 5-HT_7_R) and dopamine D_2_ receptor (D_2_R) in radioligand binding assays. The tested compounds displayed high selectivity over 5-HT_1A_, 5-HT_2A_, 5-HT_7_, and D_2_ receptors (Table 2). In contrast to the reference, intepirdine, the selected compounds did not show any significant affinity for 5-HT_2A_R. Thus, they might be devoid of the side effects associated with the modulation of 5-HT_2A_R, such as hallucinations, psychosis, fear, hypotension, headache, and dizziness.^28,29^

**Table 2:** Binding Data of Selected Compounds **18**, **22**, **27**, **28** and Intepirdine for 5-HT_6_, 5-HT_1A_, 5-HT_2A_, 5-HT_7_, and D_2_ Receptors

	*K*_i_ [nM]^a^				
compd	5-HT_6_R	5-HT_1A_R	5-HT_2A_R	5-HT_7_R	D_2_R
**18**	25	1366	14610	65520	6843
**22**	28	65680	15180	7063	6659
**27**	30	53670	13470	42000	7080
**28**	22	61490	8565	3528	7336
intepirdine^b^	1.4	2370	26	14230	997

a Mean *K*_i_ values (SEM ± 27%) based on three independent binding experiments.

b Data taken from ref (30).

### 3.3 Functional Profiles of Selected Compounds

The effect of compounds **18**, **22**, **27**, and **28** on adenylate cyclase was examined in 1321N1 cells expressing the 5-HT_6_R. Regardless of the type of substituent at the 2-phenyl-1*H*-pyrrole moiety and spatial isomerism, all of the tested compounds inhibited the 5-carboxamidotryptamine (5-CT)-stimulated cAMP accumulation and thus were classified as 5-HT_6_R antagonists (*K*_b_ = 6–35 nM) (Table 3).

**Table 3:** Antagonist Property of Selected Compounds in 1321N1 Cells and Their Functional Activity at 5-HT_6_R-Dependent Gs Signaling in NG108-5 Cells

compd	5-HT_6_R *K*_i_ [nM]^a^	5-HT_6_R *K*_b_ [nM]^b^	Gs signaling IC_50_ [nM]^c^	functional profile
**18**	25	35.0	170	inverse agonist
**22**	28	6.0	306	inverse agonist
**27**	30	11.4	265	inverse agonist
**28**	22	7.6	140	inverse agonist
CPPQ^d^	3	0.41		neutral antagonist
SB-271046	1.2^e^	1.95^e^	98	inverse agonist

a Mean *K*_i_ values (SEM ± 19%) based on three independent binding experiments.

b Mean *K*_b_ values (SEM ± 15%) obtained in three independent experiments in 1321N1 cells.

c Mean IC_50_ values (SEM ± 18%) obtained in three independent experiments in NG108-15 cells.

d Data taken from ref (17), where CPPQ is encoded as 14.

e Data taken from ref (31).

**Figure 3:**
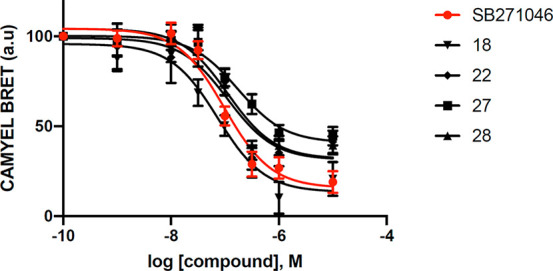
Impact of compounds **18**, **22**, **27**, and **28** and SB-271046 on basal cAMP production in NG108-15 cells transiently expressing 5-HT_6_Rs. Data are the mean ± SEM of the values obtained in three independent experiments performed in quadruplicate in different sets of cultured cells: ***P < 0.001 vs vehicle (ANOVA followed by Student–Newman–Keuls test).

As the high level of 5-HT_6_R constitutive activity was observed in *in vitro* and *in vivo* settings,^12,13^ we subsequently evaluated whether the representatives of 2-phenyl-1*H*-pyrrole-3-carboxamides (**18**, **22**, **27**, **28**) modulate agonist-independent 5-HT_6_R-operated Gs signaling. Experiments were performed in NG108-15 cells transiently expressing recombinant receptors, a cellular model exhibiting constitutively active 5-HT_6_R. In contrast to CPPQ, a reference neutral antagonist,^13,17,32^ all the tested compounds decreased basal cAMP level in a concentration-dependent manner and behaved as 5-HT_6_R inverse agonists (Figure 3). Additionally, the evaluated compounds showed potency similar to that of SB-271046, the reference 5-HT_6_R inverse agonist (Table 3). Thus, it may be concluded that the replacement of the 1*H*-pyrrolo[3,2-*c*]quinoline scaffold with the 2-phenyl-1*H*-pyrrole-3-carboxamide moiety shifted the functional activity of compounds at the Gs signaling pathway from neutral antagonism to inverse agonism and allowed us to target different active states of the 5-HT_6_R.

**Figure 4:**
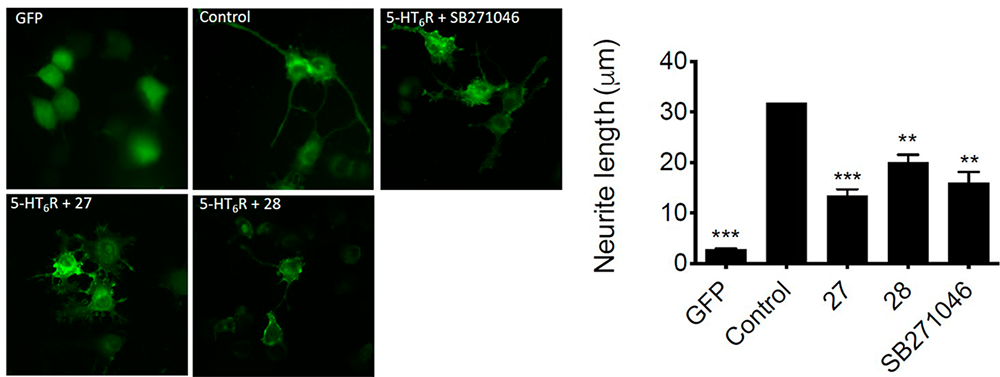
Impact of compounds **27** and **28** on Cdk5 signaling pathway. NG108-15 cells were transfected with a plasmid encoding a GFP-tagged 5-HT_6_ receptor or GFP alone. Cells expressing the receptor were exposed to DMSO (control), compounds **27** and **28**, and SB-271046 (10^–8^ M) for 24 h. The histogram shows the mean ± SEM of neurite length for each experimental condition measured from three independent experiments: ***P < 0.001 vs cells expressing GFP; ANOVA followed by Student–Newman–Keuls test.

In addition to the canonical Gs-adenylyl cyclase pathway, the 5-HT_6_R activates Cdk5 signaling in an agonist-independent manner. Preventing this activation with inverse agonists inhibits neurite growth.^10^ Compound **28** which possesses the most potent inverse agonist properties at Gs signaling and its enantiomer **27** were examined to assess their effect on Cdk5-dependent neurite growth. As shown in Figure 4, transfection of the 5-HT_6_R in NG108-15 cells results in a significant increase in neurite length as compared to green fluorescent protein (GFP) transfected cells. Both compounds **27** and **28**, applied at a concentration of 10 nM, significantly reduced neurite length in NG108-15 cells in the same way as our reference SB-271046 (with decreases of 36.9%, 57.5%, and 49.6%, respectively). Thus, these compounds might be regarded as full inverse agonists at agonist-independent 5-HT_6_R-mediated Cdk5 signaling. Of note, compound **27** displayed more significant impact on Cdk5 signaling compared to **28** and SB-271046 (Figure 4).

### 3.4 Pharmacokinetics Evaluation

Preliminary ADME and pharmacokinetics assessment play a crucial role in the optimization process of a preclinical lead compound. Thus, compounds **27** and **28** were further subjected to biotransformation studies using rat liver microsomes (RLMs). The tested compounds showed a low value of intrinsic clearance (0.82 μL/min/mg and 1.03 μL/min/mg for **27** and **28**, respectively), indicating high metabolic stability.

Subsequently, the pharmacokinetic profile of **27** and **28** was assessed in male Wistar rats after single intragastric administration at the dose of 10 mg/kg. The studied compounds were rapidly absorbed and crossed the blood–brain barrier, reaching the *C*_max_ in 5 min in both plasma and brain. Compound **27** showed higher distribution in the systemic circulation, with the brain/plasma ratio of 0.63 (Table 4). Although **28**, the *S* enantiomer of **27**, showed slightly higher affinity and antagonism at Gs signaling for the 5-HT_6_R in *in vitro* assays, the *in vivo* pharmacokinetic profile precluded its further development.

**Table 4:** Pharmacokinetic Parameters for **27** and **28**^a^

	**27**		**28**	
parameter	plasma	brain	plasma	brain
AUC_0→*t*_ [ng·min/mL]	77251	48727	203547	17335
MRT [min]	163.3	180.9	200.8	266
*t*_0.5_ [min]	117.5	250.9	297	
*C*_max_ [ng/mL][ng/g]^b^	1039.12	427.1	3498.7	51.7
*t*_max_ [min]	5	5	5	5

a Measured after *p.o.* administration of dose 10 mg/kg: *t*_0.5_, terminal half-life; AUC, area under the curve; MRT, mean residence time; *C*_max_ maximum concentration; *t*_max_, time to reach the maximum concentration.

b Concentration in brain. N = 64.

### 3.5 Extended *in Vitro* Off-Target Selectivity and Safety Profile Assessment for Compound **27**

Compound **27** was subsequently evaluated for its affinity toward several off-targets. It was found that **27** did not bind to α_1A_ adrenoreceptor (15% at 1 μM), M_1_ muscarinic (0% at 1 μM), H_1_ histaminic (9% at 1 μM), D_3_ dopamine (17% at 1 μM), and serotonin 5-HT_2C_ (10% at 1 μM) and 5-HT_3_ (2% at 1 μM) receptors; it also did not exhibit affinity for the serotonin transporter (SERT) (10% at 1 μM) and the human ether-a-go-go-related gene (*h*ERG) channel (1% at 1 μM). Therefore, compound **27** should not induce adverse effects associated with the above targets, i.e., convulsions, anxiety, psychosis, hypotension, cardiac arrhythmia, nausea, and vomiting.

**Figure 5:**
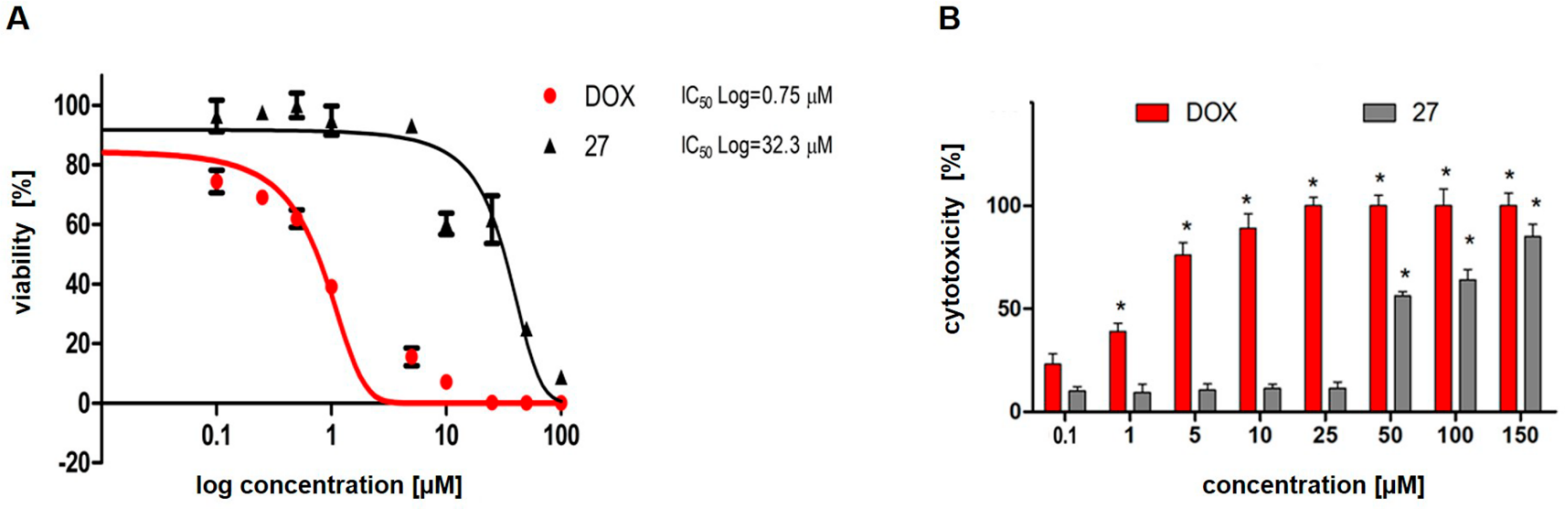
Effect of compound **27** on hepatotoxicity in HepG2 cellular model. Cells were cultured in the presence of compound **27** for 24 h. Doxorubicin (DOX) was used as reference standard. (A) MTT assay was performed to measure cellular viability (mitochondria metabolism rate). The log dose–response curves were generated in GraphPad Prism 7. Data represent the mean ± SD of three repeats. (B) LDH assay was used to study the cytotoxicity of **27**. Graph represents (percent of cells cytotoxicity) – (% of LDH release to culture medium compared to control condition) incubated with **27** in concentrations 0.1–150 μM. Data represent the mean ± SD of two repeats: *P < 0.05 (Mann–Whitney *U* test).

Considering a potential drug–drug interaction, **27** was further evaluated for its inhibitory activity on human cytochrome P450 (CYP450). Although compound **27** decreased the activity of CYP3A4 subtype (IC_50_ = 69 nM), it did not significantly influence CYP2D6. Since drug-induced liver injuries constitute a clinical issue, compound **27** was tested in the human hepatocellular carcinoma (HepG2) model to exclude its hepatotoxicity. Compound **27** did not impair the metabolic activity of cells assessed in the MTT test and did not affect the cell membrane integrity as determined in the LDH test in a wide range of concentrations (0.1–25 μM) (Figure 5). Finally, compound **27** was subjected to the Ames test to assess its potential to induce mutation in genes involved in histidine synthesis. Compound **27** was found to be non-mutagenic using *Salmonella typhimurium* TA10.

### 3.6 Behavioral Evaluation of the Procognitive Effects of Compound **27**

**Figure 6:**
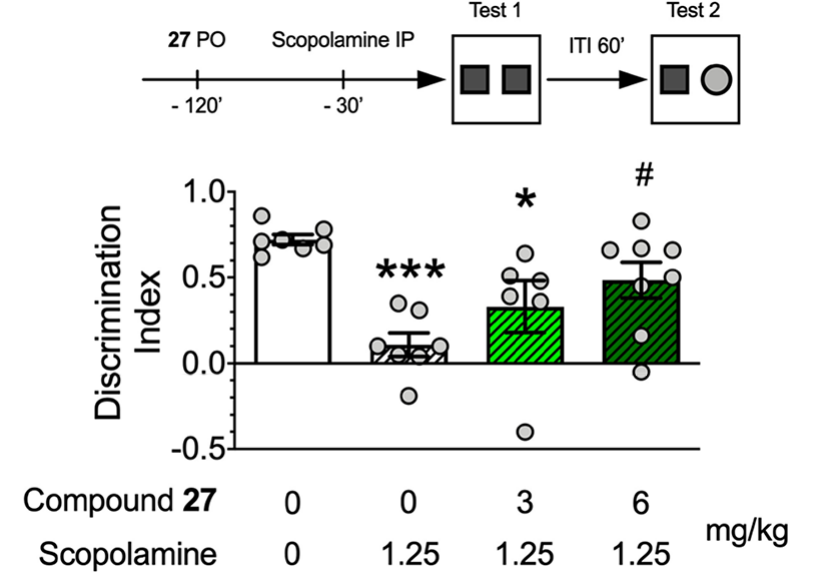
Effects of compound **27** (3 and 6 mg/kg, *p.o.*) on scopolamine-induced cognitive impairment in the novel object recognition test in rats. Data are presented as the mean ± SEM of discrimination index (DI). For vehicle, scopolamine, **27** (3 mg/kg) + scopolamine, and **27** (6 mg/kg) + scopolamine, we used 7, 7, 6, and 8 rats, respectively, summing to 28 animals: *P < 0.05; ***P < 0.001 vs vehicle; ^#^*P* < 0.05 vs scopolamine (Tukey’s multiple comparisons post-hoc test, following one-way ANOVA, *F*(3,24) = 7.30, *P* = 0.0012).

5-HT_6_R antagonists, which behave as neutral antagonists or inverse agonists, enhanced cognitive performance in animal models.^17,33,34^ Thus, the effect of compound **27** on short-term memory was investigated in the NOR test in rats treated with scopolamine.^35−37^ As expected, scopolamine-treated but not vehicle-treated rats spent significantly less time exploring the novel object than the familiar one, indicating that scopolamine abolished the ability to discriminate novel and familiar objects. Administration of scopolamine blocks muscarinic acetylcholine receptors and thus serves as a pharmacological model of cognitive decline. Compound **27** given *p.o.* at the dose of 6, but not 3 mg/kg, prevented scopolamine-induced cognitive deficits in the NOR test (Figure 6).

**Figure 7:**
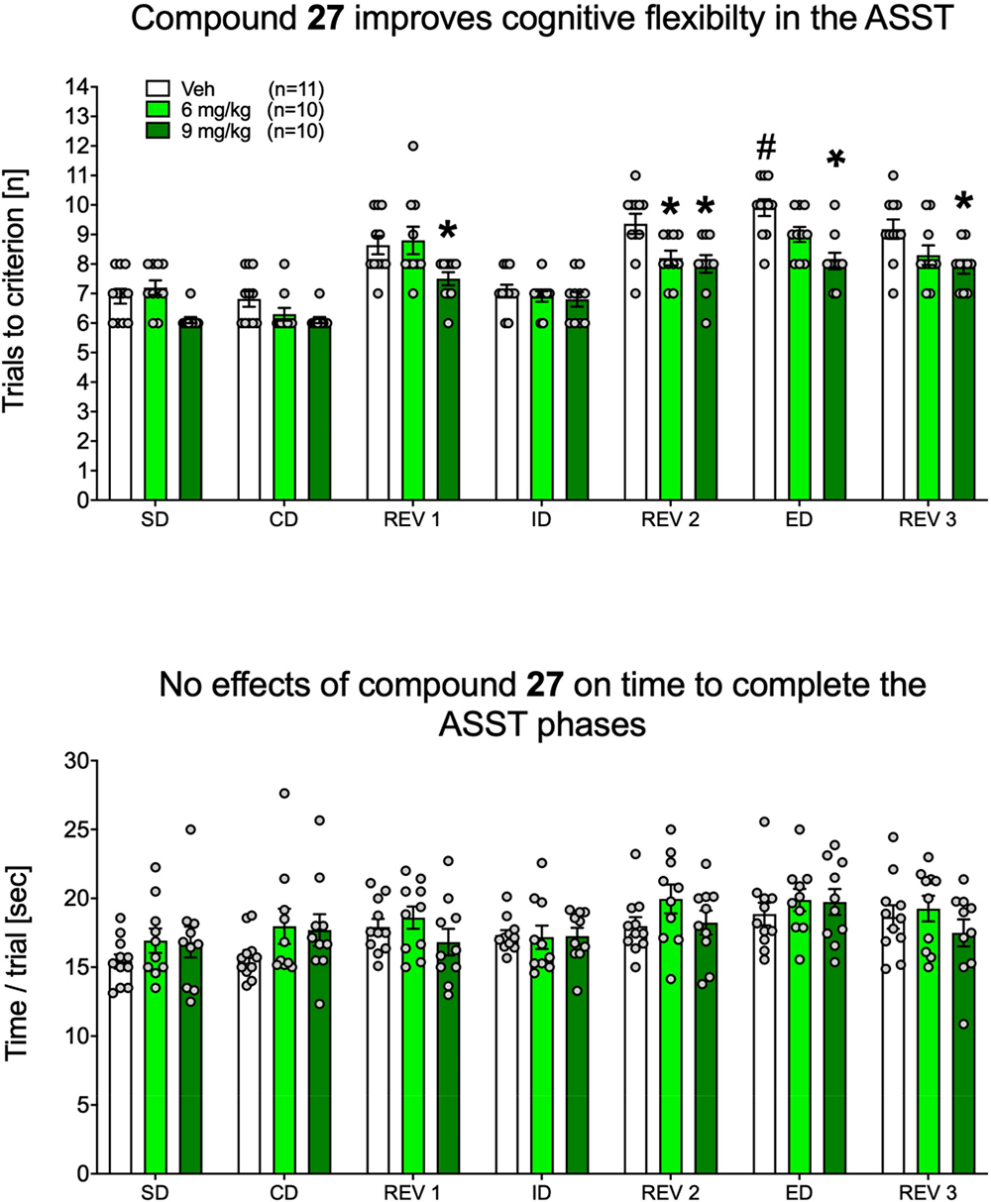
Effects of compound **27** (6 and 9 mg/kg, *p.o.*) in attentional set shifting test (ASST). Data are presented as the mean ± SEM of the trials to criterion (upper panel) and time to complete the trial during discrimination stages (lower panel). For **27** used at the doses of 0, 6, and 9 mg/kg, there were 11, 10, and 10 rats, respectively, per group, summing to 31 animals: *P < 0.05 vs respective vehicle; ^#^*P* < 0.05 vs vehicle at the intradimensional (ID) stage (Newman–Keuls post-hoc test, following significant two-way ANOVA’s stage × treatment interaction: *F*(12,168) = 1.95, *P* = 0.031). For the time to complete the trial during discrimination stages data (lower panel) two-way ANOVA’s stage × treatment interaction yielded insignificant results: *F*(12,168) = 0.945, NS.

Neuropsychiatric and neurodegenerative disorders are associated with cognitive impairments, including cognitive inflexibility, resulting in deficient adaptation to the changing conditions. These impairments are often reported in schizophrenic patients and in subjects with lesions of the prefrontal cortex.^38^ The effects of compound **27** were further examined in the ASST. This test consists of a series of two-choice discriminations, in which one of the two stimulus dimensions (e.g., the material covering the bait) is relevant and the other dimension (e.g., an odor applied to the bait container) is irrelevant for successful discrimination. The animals are required to learn the currently relevant rule and must maintain the application of that specific rule to a novel set of stimuli. This, in turn, leads to the formation of an attentional set that can be seen as “locking” the subject within an initially relevant dimension. In the crucial extra-dimensional (ED) stage, the animals must switch their attention to previously irrelevant stimulus dimension; for instance, they have to discriminate between the odors instead of the materials covering the food reward. The animal’s performance during the ED stage is regarded as an index of cognitive flexibility. Additionally, reversal learning stages examine rats’ ability to adjust responses following a change in the stimuli signifying food reward. Reduction in the number of trials to criterion at the reversal trials and at the ED stage in particular suggests an improvement in cognitive flexibility.^39^ Compound **27** administered at the dose of 9 mg/kg *p.o.* enhanced cognitive flexibility as indicated by a decreased number of trials to criterion during the ED and three reversal stages (Rev1, Rev2, and Rev3). The lower dose of compound **27** (6 mg/kg) was effective only during the Rev2 phase (Figure 7, upper panel). Additionally, compound **27** did not affect the mean time to complete the trial during any of the discrimination stages (Figure 7, lower panel).

## 4 Conclusions

The scaffold-hopping approach around pyrroloquinoline derivatives was applied to design 2-phenyl-1*H*-pyrrole-3-carboxamides as a new structural framework for developing 5-HT_6_R antagonists. SAR studies revealed the subsequent structural requirements for high affinity for the 5-HT_6_R, i.e., a fluorine atom in the *C*^4^ position at the 2-phenylpyrrole fragment, a 3-chlorophenylsulfonyl moiety in position *N*^1^, and pyrrolidine as a basic center. The *in vitro* functional activity evaluation for 5-HT_6_R-operated Gs and Cdk5 signaling allowed classification of the identified compounds as 5-HT_6_R inverse agonists. This observation is in sharp contrast to the prototypic pyrroloquinoline-based compound CPPQ, which behaved as a neutral antagonist. Selected compound **27** showed good brain distribution, no cytotoxicity, and no mutagenic activity. Therefore, it might be placed in low-risk safety space. The cognition-enhancing properties of **27** were subsequently demonstrated in the NOR test (6 mg/kg, *p.o.*) in scopolamine-induced memory decline conditions. Compound **27** also increased the cognitive flexibility mediated by the prefrontal cortex in the ASST in rats (9 mg/kg, *p.o.*). In view of the above findings, compound **27** might be regarded as a probe to study the contribution of agonist-independent 5-HT_6_R activity to neurological and neurodegenerative disorders.

## 5 Experimental Methods

### 5.1 Chemistry

#### 5.1.1 General Methods

The synthesis was carried out at ambient temperature unless indicated otherwise. Organic solvents (from Aldrich and Chempur) were of reagent grade and were used without purification. All reagents (Sigma-Aldrich, Fluorochem, Across, and TCI) were of the highest purity. Column chromatography was performed using silica gel Merck 60 (70–230 mesh ASTM).

Mass spectra were recorded on a UPLC–MS/MS system consisting of a Waters ACQUITY UPLC (Waters Corporation, Milford, MA, USA) coupled to a Waters TQD mass spectrometer. All the analyses were carried out using an Acquity UPLC BEH C18 100 × 2.1 mm^2^ column at 40 °C. A flow rate of 0.3 mL/min and a gradient of (0–100)% B over 10 min was used: eluent A, water/0.1% HCOOH; eluent B, acetonitrile/0.1% HCOOH. Retention times, *t*_R_, were given in minutes. The UPLC/MS purity of all the test compounds and key intermediates was determined to be >98%. HRMS analyses were performed on an UPLC Acquity H-class from Waters hyphenated to a Synapt G2-S mass spectrometer with a dual ESI source from Waters.

^1^H NMR and ^13^C NMR spectra were recorded at 300 and 75 MHz (Varian BB 200), 400 and 101 MHz (Bruker Avance III), or 500 and 126 MHz (JEOL JNM-ECZR500 RS1) using CDCl_3_ or CD_3_OD as solvents. Chemical shifts are given in ppm. The *J* values are reported in hertz (Hz), and the splitting patterns are designated as follows: br s (broad singlet), s (singlet), d (doublet), t (triplet), q (quartet), dd (doublet of doublets), dt (doublet of triplets), td (triplet of doublets), ddd (doublet of doublets of doublets), m (multiplet).

The synthetic procedures for intermediates **5** and **6** and final compounds (**7**–**32**) as well as characterization data for selected final compounds (**18**, **22**, **27**, **28**) are presented below. The synthesis of intermediates **1**–**4** was performed according to the previously described procedures^19,20^ and is reported in the Supporting Information together with spectroscopic data for all intermediates and remaining final compounds.

#### 5.1.2 General Procedure for Ester Hydrolysis (**5a**–**c**)

The appropriate methyl ester derivative **4** (1 equiv) was heated under reflux with the excess of 10% aqueous solution of NaOH for 3h. Then, 10% aqueous solution of HCl was added portionwise to acidic pH. The mixture was diluted with EtOAc and washed three times with water and once with brine. The organic layer was dried with Na_2_SO_4_, evaporated, and dried under vacuum.

#### 5.1.3 General Procedure for Amide Coupling (**6a**–**f**)

The carboxylic acid **5** (1 equiv) was added to a solution of 1-hydroxybenzotriazole (HOBT) (1.2 equiv) and benzotriazole-1-yl-oxy-tris(dimethylamino)phosphonium hexafluorophosphate (BOP) (1.2 equiv) in DMF, and the mixture was stirred for 30 min with triethylamine (3 equiv). Then, (*R*) or (*S*) enantiomer of 1-Boc-3-aminopyrrolidine (1.2 equiv) was added and left to react overnight. The mixture was diluted with EtOAc and washed with H_2_O and brine, dried (Na_2_SO_4_), concentrated, and purified over silica gel.

#### 5.1.4 General Procedure for the Synthesis of Final Compounds **7**–**32**

The synthesized amide **6** (1 equiv) was dissolved in CH_2_Cl_2_, and phosphazene base P_1_-*t*-Bu-tris(tetramethylene) (BTPP) (1.2 equiv) was added. The mixture was cooled down (ice bath), appropriate sulfonyl chloride (1.2 equiv) was added, and the reaction mixture was stirred for 3 h. Subsequently, the mixture was evaporated and the remaining crude product was purified on silica gel. Then, the free bases were dissolved in anhydrous ethanol and treated with 1.25 M methanolic HCl to give the final products as hydrochloride salts after evaporation.

#### 5.1.5 Characterization Data for Selected Final Compounds

##### 5.1.5.1 (*S*)-2-Phenyl-1-[(3-chlorophenyl)sulfonyl]-*N*-(pyrrolidin-3-yl)-1*H*-pyrrole-3-carboxamide (**18**)

Boc-derivative: colorless oil, 0.12 g (yield 64%) after chromatographic purification over silica gel with EtOAc/Hex (7/3, v/v); UPLC/MS purity 98%, *t*_R_ = 7.74, C_26_H_28_ClN_3_O_5_S, MW 530.04, monoisotopic mass 529.14, [M + H]^+^ 530.1.

Hydrochloride: white solid, 0.07 g (yield 66%); UPLC/MS purity 100%, *t*_R_ = 4.80, C_21_H_21_Cl_2_N_3_O_3_S, MW 466.38. ^1^H NMR (500 MHz, CD_3_OD) δ 1.72–1.81 (m, 1H), 2.09–2.18 (m, 1H), 3.02 (dd, *J* = 12.3, 4.9 Hz, 1H), 3.16–3.27 (m, 2H), 3.36 (dd, *J* = 12.3, 7.2 Hz, 1H), 4.25–4.31 (m, 1H), 6.75 (d, *J* = 3.4 Hz, 1H), 7.01–7.05 (m, 2H), 7.12 (t, *J* = 2.0 Hz, 1H), 7.28–7.34 (m, 3H), 7.39–7.46 (m, 2H), 7.57 (d, *J* = 3.4 Hz, 1H), 7.60–7.64 (m, 1H). ^13^C NMR (126 MHz, CD_3_OD) δ 29.5, 44.3, 48.9, 49.5, 110.5, 122.5, 122.9, 125.6, 127.3, 127.4, 129.0, 129.2, 130.9, 131.9, 134.4, 134.8, 135.5, 139.5, 165.1, monoisotopic mass 429.09, [M + H]^+^ 430.1; HRMS calculated for C_21_H_20_ClN_3_O_3_S 429.0914; found 430.1000.

##### 5.1.5.2 (*S*)-2-(3-Fluorophenyl)-1-[(3-chlorophenyl)sulfonyl)-*N*-(pyrrolidin-3-yl)-1*H*-pyrrole-3-carboxamide (**22**)

Boc-derivative: colorless oil, 0.10 g (yield 68%) after chromatographic purification over silica gel with EtOAc/Hex (6/4, *v/v*); UPLC/MS purity 99%, *t*_R_ = 7.97, C_26_H_27_ClFN_3_O_5_S, MW 548.03, monoisotopic mass 547.13, [M + H]^+^ 548.1.

Hydrochloride: white solid, 0.18 g (yield 76%), UPLC/MS purity 100%, *t*_R_ = 5.06, C_21_H_20_Cl_2_FN_3_O_3_S, MW 484.37. ^1^H NMR (500 MHz, CD_3_OD) δ 1.84–1.92 (m, 1H), 2.15–2.24 (m, 1H), 3.08 (dd, *J* = 12.2, 5.0 Hz, 1H), 3.19–3.27 (m, 1H), 3.31–3.42 (m, 2H), 4.26–4.33 (m, 1H), 6.74–6.79 (m, 2H), 6.82 (dt, *J* = 7.6, 1.1 Hz, 1H), 7.15–7.21 (m, 1H), 7.22 (t, *J* = 1.9 Hz, 1H), 7.31 (td, *J* = 7.9, 6.0 Hz, 1H), 7.35–7.38 (m, 1H), 7.45 (t, *J* = 7.9 Hz, 1H), 7.60 (d, *J* = 3.7 Hz, 1H), 7.64–7.68 (m, 1H). ^13^C NMR (126 MHz, CD_3_OD) δ 29.4, 44.4, 49.0, 49.5, 110.3, 115.9 (d, *J* = 21.7 Hz), 118.8 (d, *J* = 22.9 Hz), 122.8, 123.0, 125.6, 127.3, 127.8 (d, *J* = 3.0 Hz), 128.9 (d, *J* = 8.5 Hz), 131.0, 131.2, 134.1, 134.5, 134.9, 139.4, 161.8 (d, *J* = 245.1 Hz), 164.8, monoisotopic mass 447.08, [M + H]^+^ 448.1; HRMS calculated for C_21_H_19_ClFN_3_O_3_S 448.0820; found 448.0898.

##### 5.1.5.3 (*R*)-2-(4-Fluorophenyl)-1-[(3-chlorophenyl)sulfonyl]-*N*-(pyrrolidin-3-yl)-1*H*-pyrrole-3-carboxamide (**27**)

Boc-derivative: colorless oil, 0.12 g (yield 77%) after chromatographic purification over silica gel with EtOAc/Hex (6/4, v/v); UPLC/MS purity 100%, *t*_R_ = 7.92, C_26_H_27_ClFN_3_O_5_S, MW 548.03, monoisotopic mass 547.13, [M + H]^+^ 548.1.

Hydrochloride: white solid, 0.12 g (yield 80%), UPLC/MS purity 99%, *t*_R_ = 4.93, C_21_H_20_Cl_2_FN_3_O_3_S, MW 484.37. ^1^H NMR (500 MHz, CD_3_OD) δ 1.85–1.94 (m, 1H), 2.17–2.26 (m, 1H), 3.11 (dd, *J* = 12.2, 5.0 Hz, 1H), 3.22–3.29 (m, 1H), 3.33–3.44 (m, 2H), 4.30–4.38 (m, 1H), 6.81 (d, *J* = 3.7 Hz, 1H), 7.06 (d, *J* = 7.2 Hz, 4H), 7.18 (t, *J* = 1.7 Hz, 1H), 7.36–7.39 (m, 1H), 7.47 (t, *J* = 8.2 Hz, 1H), 7.59 (d, *J* = 3.4 Hz, 1H), 7.67 (ddd, *J* = 8.1, 2.1, 1.0 Hz, 1H). ^13^C NMR (126 MHz, CD_3_OD) δ 29.5, 44.3, 48.9, 49.5, 110.4, 114.2 (d, *J* = 21.7 Hz), 122.6, 123.0, 125.1 (d, *J* = 3.6 Hz), 125.5, 127.3, 131.0, 134.0 (d, *J* = 9.0 Hz), 134.5, 134.6, 134.9, 139.4, 163.4 (d, *J* = 250.5 Hz, 164.9, monoisotopic mass 447.08, [M + H]^+^ 448.1; HRMS calculated for C_21_H_19_ClFN_3_O_3_S 448.0820; found 448.0896.

##### 5.1.5.4 (*S*)*-*2-(4-Fluorophenyl)-1-[(3-chlorophenyl)sulfonyl]-*N*-(pyrrolidin-3-yl)-1*H*-pyrrole-3-carboxamide (**28**)

Boc-derivative: colorless oil, 0.451 g (yield 61%) after chromatographic purification over silica gel with EtOAc/Hex (6/4, v/v); UPLC/MS purity 100%, *t*_R_ = 7.91, C_26_H_27_ClFN_3_O_5_S, MW 548.03, monoisotopic mass 547.13, [M + H]^+^ 548.1.

Hydrochloride: white solid, 0.321 g (yield 83%), UPLC/MS purity 100%, *t*_R_ = 4.93, C_21_H_20_Cl_2_FN_3_O_3_S, MW 484.37. ^1^H NMR (500 MHz, CD_3_OD) δ 1.85–1.93 (m, 1H), 2.17–2.26 (m, 1H), 3.11 (dd, *J* = 12.2, 5.0 Hz, 1H), 3.22–3.29 (m, 1H), 3.33–3.44 (m, 2H), 4.31–4.37 (m, 1H), 6.81 (d, *J* = 3.7 Hz, 1H), 7.07 (d, *J* = 7.2 Hz, 4H), 7.19 (t, *J* = 2.0 Hz, 1H), 7.36–7.39 (m, 1H), 7.48 (t, *J* = 8.0 Hz, 1H), 7.60 (d, *J* = 3.4 Hz, 1H), 7.67 (ddd, *J* = 8.0, 3.4, 0.9 Hz, 1H). ^13^C NMR (126 MHz, CD_3_OD) δ 29.5, 44.3, 48.9, 49.5, 110.4, 114.2 (d, *J* = 22.3 Hz), 122.60, 123.0, 125.1 (d, *J* = 3.6 Hz), 125.6, 127.3, 131.0, 134.0 (d, *J* = 8.4 Hz), 134.5, 134.6, 134.9, 139.4, 163.4 (d, *J* = 249.9 Hz), 164.9, monoisotopic mass 447.08, [M + H]^+^ 448.1; HRMS calculated for C_21_H_19_ClFN_3_O_3_S 448.0820; found 448.0898.

### 5.2 Molecular Docking

The 5-HT_6_R homology models were obtained according to the procedure described before on the β_2_ receptor template (PDB code 4LDE) and successfully used in our earlier studies of different groups of 5-HT_6_R ligands simulations.^24,40^ The three-dimensional structures of the ligands were prepared using LigPrep version 3.6 [Schrödinger Release 2020-4: LigPrep, Schrödinger, LLC, New York, NY, 2020], and the appropriate ionization states at pH = 7.0 ± 0.5 were assigned using Epik version 3.4 [Schrödinger Release 2020-4: Epik, Schrödinger, LLC, New York, NY, 2020]. Protein Preparation Wizard was used to assign bond orders and appropriate amino acid ionization states and to check for steric clashes. The receptor grid was generated by centering the grid box with a size of 12 Å on D3.32. Automated flexible docking was performed using Glide version 7.0 at the SP level [Schrödinger Release 2020-4: Glide, Schrödinger, LLC, New York, NY, 2020].

### 5.3 *In Vitro* Pharmacological Evaluation

#### 5.3.1 Radioligand Binding Assays

All experiments were carried out according to the previously published procedures.^41−43^ HEK293 cells stably expressing human 5-HT_1A_, 5-HT_6_, 5-HT_7b_, and D_2L_ receptors (prepared with the use of Lipofectamine 2000) or CHO-K1 cells with plasmid containing the sequence coding for the human serotonin 5-HT_2A_ receptor (PerkinElmer) were maintained at 37 °C in a humidified atmosphere containing 5% CO_2_ and grown in Dulbecco’s modified Eagle’s medium containing 10% dialyzed fetal bovine serum and 500 μg/mL G418 sulfate. For membrane preparation, cells were cultured in 150 cm^2^ flasks, grown to 90% confluence, washed twice with prewarmed to 37 °C phosphate buffered saline (PBS), and centrifuged (200*g*) in PBS containing 0.1 mM EDTA and 1 mM dithiothreitol. Prior to membrane preparation, pellets were stored at −80 °C. Cell pellets were thawed and homogenized in 10 volumes of assay buffer using an Ultra Turrax tissue homogenizer and centrifuged twice at 35 000*g* for 15 min at 4 °C, with incubation for 15 min at 37 °C between the centrifugations. The composition of the assay buffers was experimentally selected to achieve the maximum signal window (more details in Supporting Information). All assays were carried out in a total volume of 200 μL in 96-well plates for 1 h at 37 °C except 5-HT_1A_R and 5-HT_2A_R which were incubated at room temperature and 27 °C, respectively. The process of equilibration was terminated by rapid filtration through Unifilter plates with a 96-well cell harvester, and radioactivity retained on the filters was quantified on a Microbeta plate reader (PerkinElmer, USA). For displacement studies the assay samples contained the following as radioligands (PerkinElmer, USA): 2.5 nM [^3^H]-8-OH-DPAT (135.2 Ci/mmol) for 5-HT_1A_R; 1 nM [^3^H]-ketanserin (53.4 Ci/mmol) for 5-HT_2A_R; 2 nM [^3^H]-LSD (83.6 Ci/mmol) for 5-HT_6_R; 0.8 nM [^3^H]-5-CT (39.2 Ci/mmol) for 5-HT_7_R or 2.5 nM [^3^H]-raclopride (76.0 Ci/mmol) for D_2L_R. Nonspecific binding was defined in the presence of 10 μM 5-HT in 5-HT_1A_R and 5-HT_7_R binding experiments, whereas 20 μM mianserin, 10 μM methiothepine, and 10 μM haloperidol were used in 5-HT_2A_R, 5-HT_6_R, and D_2L_R assays, respectively. Each compound was tested in triplicate at seven concentrations (10^–10^–10^–4^ M). The inhibition constants (*K*_i_) were calculated from the Cheng–Prusoff equation.^44^

Additionally, the affinity of compound **27** at the adrenergic α_1A_, muscarinic M_1_, histaminergic H_1_, dopaminergic D_3_, serotoninergic 5-HT_2C_, 5-HT_3_ receptors, serotonin transporter (SERT), and the human ether-a-go-go-related gene (hERG) channel were evaluated in Eurofins Cerep. The results were expressed as the % inhibition at 1 μM according to experimental protocols described online at https://www.eurofins.com/.

#### 5.3.2 Determination of cAMP Production in 1321N1 Cells

The properties of compounds **18**, **22**, **27**, **28** to inhibit cAMP production induced by 5-CT (1000 nM), a 5-HT_6_R agonist, were evaluated. Compounds were tested in triplicate at eight concentrations (10^–11^–10^–4^ M). The level of cAMP was measured using frozen recombinant 1321N1 cells expressing the human serotonin 5-HT_6_R (PerkinElmer). Total cAMP was measured using the LANCE cAMP detection kit (PerkinElmer), according to the manufacturer’s recommendations. For quantification of cAMP levels, 2000 cells/well (5 μL) were incubated with a mixture of compounds (5 μL) for 30 min at room temperature in 384-well white opaque microtiter plate. After incubation, the reaction was stopped and cells were lysed by the addition of 10 μL of working solution (5 μL of Eu-cAMP and 5 μL of ULight-anti-cAMP) for 1 h at room temperature. Time-resolved fluorescence resonance energy transfer (TR-FRET) was detected by an Infinite M1000 Pro (Tecan) using instrument settings from LANCE cAMP detection kit manual. *K*_b_ values were calculated from Cheng–Prusoff equation specific for the analysis of functional inhibition curves: *K*_b_ = IC_50_/(1 + *A*/EC_50_) where *A* represents agonist concentration, IC_50_ the concentration of antagonist producing a 50% reduction in the response to agonist, and EC_50_ the agonist concentration which causes a half of the maximal response.^44^

#### 5.3.3 Determination of cAMP Production in NG108-15 Cells

NG108-15 cells were grown in Dulbecco’s modified Eagle’s medium (DMEM) supplemented with 10% dialyzed fetal calf serum, 2% hypoxanthine/aminopterin/thymidine (Life Technologies), and antibiotics. cAMP measurement was performed in cells transiently expressing 5-HT_6_R using the bioluminescence resonance energy transfer (BRET) sensor for cAMP, CAMYEL (cAMP sensor using YFP-Epac-RLuc).^45^ NG108-15 cells were cotransfected in suspension with 5-HT_6_R (0.5 μg DNA/million cells) and CAMYEL constructs (1 μg DNA/million cells), using Lipofectamine 2000 according to the manufacturer’s protocol, and plated in white 96-well plates (Greiner) at a density of 50 000 cells per well. After 24 h of transfection, cells were washed with PBS containing calcium and magnesium. To test the inverse agonist properties of compounds **18**, **22**, **27**, **28** and SB-271046, cells were treated with vehicle or with the tested compound at a concentration ranging from 0.1 nM to 10 μM. Coelanterazine H (Molecular Probes) was added at a final concentration of 5 μM and left at room temperature for 5 min. BRET was measured using a Mithras LB 940 plate reader (Berthold Technologies). Expression of 5-HT_6_R in NG108-15 cells induced a strong decrease in CAMYEL BRET signal, compared with cells transfected with an empty vector instead of the plasmid encoding the 5-HT_6_R. This decrease in CAMYEL BRET signal was thus used as an index of 5-HT_6_R constitutive activity at Gs signaling.

#### 5.3.4 Impact of Compounds on Neurite Growth

NG108-15 cells were transfected with plasmids encoding either cytosolic GFP or a GFP-tagged 5-HT_6_R in suspension using Lipofectamine 2000 (Life Technologies) and plated on glass coverslips. Six hours after transfection, cells were treated with either DMSO (control), **27**, and **28** or SB-271046 (10^–8^ M) for 24 h. Cells were fixed in 4% paraformaldehyde (PFA) supplemented with 4% sucrose for 10 min. PFA fluorescence was quenched by incubating the cells in PBS containing 0.1 M glycine, prior to mounting in Prolong Gold antifade reagent (Thermo Fisher Scientific). Cells were imaged using an AxioImagerZ1 microscope equipped with epifluorescence (Zeiss), using a 20× objective for cultured cells, and neurite length was assessed using the Neuron J plugin of the ImageJ software (NIH).

### 5.4 Pharmacokinetics Evaluation

#### 5.4.1 *In Vitro* Metabolic Stability Study

Metabolic stability of compounds **27** and **28** was analyzed using incubation systems composed of tested compound (10 μM), rat liver microsomes (RLMs, microsomes from rat male liver, pooled; 0.4 mg/mL; Sigma-Aldrich), NADPH-regenerating system (NADP+, glucose-6-phosphate, and glucose-6-phosphate dehydrogenase in 100 mM potassium buffer, pH 7.4; all from Sigma-Aldrich) and potassium buffer, pH 7.4. Stock solution of tested compounds was prepared in methanol (the final methanol concentration in incubation mixture does not exceed 0,1%). First, all samples containing incubation mixture (without NADPH-regenerating system) were preincubated in thermoblock at 37 °C for 10 min. Then reaction was initiated by the addition of NADPH-regenerating system. In control samples NADPH-regenerating system was replaced with potassium buffer. Probes were incubated for 30 and 60 min at 37 °C. After addition of internal standard (pentoxifylline, 10 μM) biotransformation process was stopped by addition of perchloric acid. Next, samples were centrifuged and supernatants were analyzed using UPLC/MS (Waters Corporation, Milford, MA). All experiments were run in duplicates. Half-life time was evaluated using nonlinear regression model using GraphPad Prism software, and intrinsic clearance was calculated from the equation Cl_int_ = (volume of incubation [μL]/protein in the incubation [mg])0.693/*t*_1/2_.^46^

#### 5.4.2 *In Vivo* Pharmacokinetic Study

##### 5.4.2.1 Instrumentation

For LC–MS/MS analyses, an HPLC Nexera system (Shimadzu, Kyoto, Japan) combined with the triple quadrupole API 3200 mass spectrometer (SCIEX, Framingham, MA, USA) interfaced via an electrospray source and controlled by Analyst software version 1.5.2 (SCIEX, Framingham, MA, USA) was applied.

##### 5.4.2.2 LC/MS/MS Analyses

The chromatographic separation was performed on Acclaim Polar Advantage II (3.0 mm × 74 mm, 3 μm, 120A, Dionex, USA) analytical column using the mobile phase composed of eluent A, HPLC grade acetonitrile acidified with 0.1% (v/v) formic acid, and eluent B, HPLC grade water with 0.1% (v/v) formic acid. The elution gradient started with 90% of eluent B, increasing to 90% of eluent A over 5 min, returned to 90% of eluent B over 5 min, and maintained at 90% of eluent B for 5 min. The mobile phase flow rate was set at 400 μL/min. The injection volume was 20 μL, and the total time of analysis was 15 min. The temperatures of the column thermostat and the autosampler were set at 40 and 10 °C, respectively.

For increased sensitivity and selectivity, the MS/MS data acquisition was performed in the selected reaction monitoring (SRM) mode. The ions measured were *m*/*z* 585.1 (Q1) and *m*/*z* 516.2 (Q3) for compounds **27** and **28** and *m*/*z* 305 (Q1) and *m*/*z* 248 (Q3) for IS. The quantification was done via peak area ratio.

The optimized MS/MS experimental conditions were as follows: ion spray voltage, 5500 V; source temperature, 300 °C; gas 1 pressure, 20 psi; gas 2 pressure, 20 psi; curtain gas pressure, 20 psi; collision gas pressure, 12 psi.

The developed method was validated according to validation procedures, parameters, and acceptance criteria based on FDA and EMA guidelines for bioanalytical method validation.^47,48^

##### 5.4.2.3 Sample Preparation

Protein precipitation with acetonitrile was used for purification of plasma and brain homogenates. The whole brain was homogenized using an electric tissue homogenizer in an appropriate amount of phosphate buffer (pH 7.4) in 1:2.5 ratio. Thereafter, a volume of 100 μL of plasma or brain homogenate was transferred to 2 mL Eppendorf tubes, and a 5 μL aliquot of the internal standard (IS, PH002437, Merck, Darmstadt, Germany) in working solution (5 μg/mL) was added and vortex-mixed for 10 s. Thereafter, 200 μL of acetonitrile was added and mixed for 20 min, followed by centrifugation (28 672*g*) for 10 min at 4 °C. The supernatant was transferred to chromatographic vials, and 20 μL was injected into the analytical column.

##### 5.4.2.4 Animals and Ethical Statement

A group of 64 male, 8-week-old, Wistar strain outbred rats weighing between 200 and 220 g each were purchased from the Animal House at the Faculty of Pharmacy, Jagiellonian University Medical College, Krakow (Poland) and housed in standard polycarbonate cages, in groups of four animals per cage. Environmental conditions during the study were constant: relative humidity 50–60%, temperature 22 ± 2 °C, normal 12 h light–dark cycle (7 a.m. to 7 p.m. light). Standard rodent chow and water were available *ad libitum*. Compounds **27** and **28** were dissolved in saline and administered by intragastric gavage at a dose of 10 mg/kg, and the animals were sacrificed at specific time-points: 0 min, predose (*n* = 4), 5 min (*n* = 4), 15 min (*n* = 4), 30 min (*n* = 4), 60 min (*n* = 4), 120 min (*n* = 4), 240 min (*n* = 4), and 480 min (*n* = 4) after administration. Animals were deeply anaesthetized by ip injections of 50 mg/kg ketamine plus 8 mg/kg xylazine before sacrifice. First, the blood was collected into heparinized tubes, and the blood samples were centrifuged at 1000*g* for 10 min to obtain plasma. Following the animals’ euthanasia, the whole brain was collected. The plasma and brain samples were immediately frozen at −80 °C for future analysis. All experimental procedures were carried out in accordance with EU Directive 2010/63/EU and approved by the I Local Ethics Committee for Experiments on Animals of the Jagiellonian University in Krakow, Poland (No. 83/2018).

### 5.5 Extended *in Vitro* Off-Target Selectivity and Safety Profile Assessment for Compound **27**

#### 5.5.1 Inhibition of Cytochrome P450: CYP3A4 and CYP2D6

The luminescent CYP3A4 P450-Glo and CYP2D6 P450-Glo assays and protocols were provided by Promega (Madison, WI, USA). The P450-Glo systems based on the conversion proluciferins, derivatives of beetle luciferin [(4S)-4,5-dihydro-2-(6′-hydroxy-2′-benzothiazolyl)-4-thiazolecarboxylic acid] into d-luciferin were catalyzed by respective CYP isoform. d-Luciferin is formed and detected in a second reaction with the luciferin detection reagent (LDR). The amount of light produced in the second reaction is proportional to CYP activity.

The stock solutions (10 mM) of the references ketoconazole (KE), quinidine (QD), and examined compounds were performed in DMSO. The 4× concentrated dilutions were prepared before the assays. The enzymatic reactions were conducted in white polystyrene, flat-bottom Nunc MicroWell 96-well microplates (Thermo Scientific, Waltham, MA USA).

The CYPs, proluciferin, and examined compounds (25 μL/well) were preincubated first for 5 min, and next the NADPH regeneration system was added (25 μL) to start the reaction. The final concentrations of KE and examined compounds were in range from 0.01 μM to 25 μM. The final concentrations of QD were from 0.001 to 10 μM. The control reactions for measuring the 100% of CYPs activity and minus-CYP negative control reactions for measure background luminescence were also prepared. The microplate was incubated in room temperature for 30 min (CYP3A4) or 45 min (CYP2D6). Finally, LDR was added (50 μL/well), and after 20 min of incubation in room temperature the luminescence signal was measured with a microplate reader (EnSpire, PerkinElmer) in luminescence mode. The signal produced by CYPs without the presence of compounds was considered as 100% of CYP activity. The IC_50_ values and were calculated using GraphPad Prism 5 software.

#### 5.5.2 Assessment of Hepatotoxic Activity

##### 5.5.2.1 Cell Culture

Human hepatocellular carcinoma cells (HepG2) were cultured using standard procedures (protocol from ATCC). Cells were cultured in Eagle’s minimum essential medium (EMEM) in flasks with an area of 25 cm^2^ (Falcon), supplemented with 10% of fetal bovine serum (FBS, Life Technologies) with the addition of 100 IU/mL penicillin (Sigma-Aldrich) and 100 μg/mL streptomycin (Sigma-Aldrich) and incubated at 37 °C in a humidified atmosphere with 5% CO_2_.

##### 5.5.2.2 LDH Assay

Cells were seeded at density 2 × 10^4^ cells/per well in 95-well plates. After 24 h, compound **27** and reference standard DOX (highly cytotoxic agent) were added to final concentrations of 0.1–150 μM. After 24 h incubation, plates were centrifuged (200*g*, 2 min) and 50 μL of the supernatant was transferred into the corresponding 96-well plate. Subsequently, 50 μL of LDH-reaction mixture prepared according to the manufacturer’s instructions (Invitrogen) was added to each well. Incubation was conducted in darkness for 30 min at room temperature. Next, stop solution was added and absorbance was measured at 490 nm (A490) using a plate reader (Spectra Max iD3, Molecular Devices). Cytotoxicity was determined as follows: cytotoxicity (%) = [(compound LDH activity – spontaneous LDH activity)/(maximum LDH activity – spontaneous LDH activity)] × 100. The maximum LDH activity was prepared by treating cells with lysis buffer. The medium used in the LDH assay contained 1% FBS. Two independent experiments were performed for each condition.

##### 5.5.2.3 MTT Assay

The MTT assay was used to determine the viability of HepG2 cells incubated in the presence of compound **27** and DOX. Cells were seeded at a density of 2 × 10^4^ in 96-well plates. Following overnight culture, the cells were treated with with **27** and DOX in concentration range 0.1–150 μM for 24 h. Following cell exposure to each compound 10 μL of MTT reagent (Sigma-Aldrich) was added to each well. After 4 h of incubation (37 °C, 5% CO_2_) the culture medium was aspirated and formazan crystals were dissolved in 100 μL of DMSO. Then, optical density (OD) at 570 nm was determined on a plate reader (Spectra Max iD3, Molecular Devices). Each individual experiment was repeated at least three times.

#### 5.5.3 Evaluation of the Mutagenic Potential: AMES Test

Ames microplate fluctuation protocol (MPF) assay was performed with *Salmonella typhimurium* strain TA100, enabling the detection base substitution mutations. Bacterial strain and exposure and indicator medium were purchased from Xenometrix AG (Allschwil, Switzerland). The mutagenic potential of tested structures was evaluated by incubation of bacteria, incapable of producing histidine, with particular concentration of test compounds for 90 min in exposure medium, containing limited amount of histidine. The occurrence of reversion events to histidine prototrophy was observed as a growth of bacteria in the indicator medium without histidine after 48 h of incubation in room temperature. Bacterial growth in 384-well plates was visualized by color change of medium from violet to yellow due to addition of pH indicator dye. Compound was classified as mutagenic, if the fold increase in number of positive wells over the medium control baseline was greater than 2.0. The solvent control baseline was defined as the mean number of positive wells in the negative control sample, increased by one standard deviation. 1% DMSO in media was used as a negative and 4-nitroquinoline *N*-oxide (NQNO) as positive control. The experiment was performed in triplicate. Fraction S9 was not added.

### 5.6 *In Vivo* Pharmacological Evaluation of the Cognitive Effects of the Compound **27**: Animals and the Ethical Statement

The experiments were conducted in accordance with the NIH Guide for the Care and Use of Laboratory Animals and were approved by the II Local Ethics Committee for Animal Experiments, Maj Institute of Pharmacology. Male inbred Sprague-Dawley rats (Charles River, Germany) weighing ∼250 g at the arrival were housed in the standard laboratory cages, under standard colony A/C controlled conditions: room temperature 21 ± 2 °C, humidity (40–50%), 12 h light/dark cycle (lights on, 06:00) with *ad libitum* access to food (unless stated otherwise) and water. Rats were allowed to acclimatize for at least 7 days before the start of the experimental procedure. During this week animals were handled at least 3 times. Behavioral testing was carried out during the light phase of the light/dark cycle. At least 1 h before the start of the experiment, rats were transferred to the experimental room for acclimation.

#### 5.6.1 Novel Object Recognition (NOR) Test under Scopolamine-Induced Memory Decline

The experiments were performed according to the previously reported procedures.^35−37^ Twenty-eight rats were tested in a dimly lit (25 Lx) “open field” apparatus made of a dull gray plastic measuring (66 cm × 56 cm × 30 cm). After each measurement, the floor was cleaned and dried.

##### 5.6.1.1 Drug Treatment in the NOR Test

Scopolamine hydrobromide used to attenuate learning was purchased from Sigma-Aldrich (Germany). Scopolamine and the compound **27** were solubilized in distilled water and then administered at the dose of 1.25 mg/kg (ip) and 3–6 mg/kg (po) 30 and 120 min before familiarization phase (T1), respectively. The choice of scopolamine dose was based on our earlier report;^37^ the doses of the **27** compound were selected based on the PK study.

##### 5.6.1.2 NOR Test Experimental Procedure and Statistics

The procedure consisted of habituation to the arena (without any objects) for 5 min, 24 h before the test, and test session comprised two trials separated by an inter trial interval (ITI). For scopolamine-induced memory impairment paradigm, 1 h ITI was chosen. During the first trial (familiarization, T1) two identical objects (A1 and A2) were presented in opposite corners, approximately 10 cm from the walls of the open field. In the second trial (recognition, T2) one of the objects was replaced by a novel one (A = familiar and B = novel). Both trials lasted 3 min, and animals were returned to their home cage after T1. The objects used were the glass beakers filled with the gravel and the plastic bottles filled with the sand. The heights of the objects were comparable (∼12 cm), and the objects were heavy enough not to be displaced by the animals. The sequence of presentations and the location of the objects were randomly assigned to each rat. The animals explored the objects by looking, licking, sniffing, or touching the object while sniffing but not when leaning against, standing, or sitting on the object. Any rat spending less than 5 s exploring the two objects within 3 min of T1 or T2 was eliminated from the study. Exploration time of the objects and the distance traveled were measured using the Any-maze video tracking system. On the basis of exploration time (E) of two objects during T2, discrimination index (DI) was calculated according to the formula: DI = (EB – EA)/(EA + AB).

Data were analyzed using one-way ANOVA with treatment as between-subject factor. As a post-hoc, we used Tukey’s multiple comparisons post-hoc test. Statistical significance was set at *P* < 0.05. Statistics was performed with Prism 9.0 for Mac.

#### 5.6.2 Attentional Set Shifting Task (ASST)

Thirty-two male Sprague-Dawley rats were group housed with a mild food restriction (17 g of food pellets per day) for at least 1 week prior to training.

##### 5.6.2.1 ASST Apparatus

Testing was conducted in a Plexiglas apparatus (length × width × height: 38 cm × 38 cm × 17 cm) with the grid floor and wall dividing half of the length of the cage into two sections. During testing, one ceramic digging pot (internal diameter of 10.5 cm and a depth of 4 cm) was placed in each section. Each pot was defined by a pair of cues along with two stimulus dimensions. To mark each pot with a distinct odor, 5 μL of a flavoring essence (Dr Oetker, Poland, or The Body Shop, U.K.) was applied to a piece of blotting paper fixed to the external rim of the pot immediately prior to use. A different pot was used for each combination of digging medium and odor; only one odor was ever applied to a given pot. The bait (one-half of a Honey Nut Cheerios, Nestle) was placed at the bottom of the “positive” pot and buried in the digging medium. A small amount of powdered Cheerios was added to the digging media to prevent the rat from trying to detect the buried reward by its smell.

##### 5.6.2.2 ASST Procedure

As described previously,^49^ the procedure lasted 3 days for each rat.

Day 1, Habituation. Rats were habituated to the testing area and trained to dig in the pots filled with sawdust to retrieve the food reward. Rats were transported from the housing facility to the testing room where they were presented with one unscented pot (filled with several pieces of Cheerios) in their home cages. After the rats had eaten the Cheerios from the home cage pot, they were placed in the apparatus and given three trials to retrieve the reward from both of the sawdust-filled baited pots. With each exposure, the bait was covered with an increasing amount of sawdust. Animals that did not dig for a food reward were subjected again to the training on the next day. If a rat did not start to dig in three daily sessions, it was excluded from an experiment.

Day 2, Training. Rats were trained on a series of simple discriminations (SDs) to a criterion of six consecutive correct trials. For these trials, rats had to learn to associate the food reward with an odor cue (e.g., arrack vs orange, both pots filled with sawdust) and/or a digging medium (e.g., plastic balls vs pebbles, no odor). All rats were trained using the same pairs of stimuli. The positive and negative cues for each rat were presented randomly and equally. These training stimuli were not used again in later testing trials.

Day 3, Testing. Rats performed a series of discriminations in a single test session. An incorrect choice was recorded as an error. Digging was defined as any distinct displacement of the digging media with either the paw or the nose; the rat could investigate a digging pot by sniffing or touching without displacing material. Experiments were performed by an experimenter blinded to the treatment group. Testing was continued at each phase until the rat reached the criterion of six consecutive correct trials, after which testing proceeded to the next phase. If a rat does not make either a correct or an incorrect response during any trials of the ASST within 5 min, the trial was reinitiated after a 10 min break. If the rat was still not responding, the test was discontinued and the rat was excluded from the data analysis.

In the simple discrimination involving only one stimulus dimension, the pots differed along one of two dimensions (e.g., digging medium). For the compound discrimination (CD), the second (irrelevant) dimension (i.e., odor) was introduced but the correct and incorrect exemplars of the relevant dimension remained constant. For the reversal of this discrimination (Rev1), the exemplars and relevant dimension were unchanged, but the previously correct exemplar was now incorrect and vice versa. The ID shift was then presented, comprising new exemplars of both the relevant and irrelevant dimensions with the relevant dimension remaining the same as previously. The ID discrimination was then reversed (Rev2) so that the formerly positive exemplar became the negative one. For the ED shift, a new pair of exemplars was again introduced, but this time a relevant dimension was also changed. Finally, the last phase was the reversal (Rev3) of the ED discrimination.

The exemplars were always presented in pairs and varied so that only one animal within each treatment group received the same combination. The assignment of each exemplar in a pair as being positive or negative at a given phase, and the left–right positioning of the pots in the test apparatus on each trial were randomized.

##### 5.6.2.3 Drugs for ASST

Compound **27** was dissolved in distilled water and was given 120 min before ED phase (i.e., 90 min before first stage of ASST test at the doses of 0, 6, and 9 mg/kg, po). The choice of **27** compound doses was based on the PK study. The drug or vehicle (distilled water) was administered at a volume of 1 mL/kg of body weight.

##### 5.6.2.4 Statistics for ASST

As the main cognitive measure, the number of trials required to achieve the criterion of six consecutive correct responses (i.e., trials to criterion, TTC) was recorded for each rat and for each discrimination phase of the ASST. Additionally, we analyzed the mean time to complete a single trial in a given discrimination stage to examine nonspecific effects of the compound **27**.

Data were analyzed using mixed design ANOVAs with treatment as between-subject factor and discrimination phase (SD, CD, Rev1, etc.) as a repeated measure. As a post-hoc, we used Newman–Keuls test. Statistical significance was set at *P* < 0.05. The statistical analyses were performed using Statistica 12.0 for Windows.

##### 5.6.2.5 Study Limitations

In the experiments examining the cognitive effects of **27** compound, we used only two doses (3 and 6 mg/kg in the NOR test and 6 and 9 mg/kg in the ASST test).

## Supporting Information Available

Synthetic procedures for intermediates **1**–**4**; characterization data for all intermediates and final compounds excluded from the main manuscript; evaluation of CYP450 inhibition and mutagenic properties (Ames test) (PDF)
