# Supplementary material for: 2-Phenyl-1H-pyrrole-3-carboxamide as a New Scaffold for Developing 5-HT6 Receptor Inverse Agonists with Cognition-Enhancing Activity
Source: ACS Chem Neurosci. 2021 Mar 11;12(7):1228–40. doi: 10.1021/acschemneuro.1c00061 (PMC8041276; doi:10.1021/acschemneuro.1c00061)
Supplement: Supplementary file 1 — cn1c00061_si_001.pdf [file cn1c00061_si_001.pdf]

Supporting Information

2-Phenyl-1*H*-pyrrole-3-carboxamide as a New Scaffold for Developing 5-HT$_6$ Receptor Inverse Agonists with Cognition-enhancing Activity

Marcin Drop,[a,b] Vittorio Canale,[a] Severine Chaumont-Dubel,[c] Rafał Kurczab,[d] Grzegorz Satała,[d] Xavier Bantreil,[b] Maria Walczak,[a] Paulina Koczurkiewicz-Adamczyk,[a] Gniewomir Latacz,[a] Anna Gwizdak,[a,c] Martyna Krawczyk,[d] Joanna Gołębiowska,[d] Katarzyna Grychowska,[a] Andrzej J. Bojarski,[d] Agnieszka Nikiforuk,[d] Gilles Subra,[b] Jean Martinez,[b] Maciej Pawłowski,[a] Piotr Popik,[d] Philippe Marin,[c] Frédéric Lamaty,[b] Paweł Zajdel,[a,*]

[a] *Faculty of Pharmacy, Jagiellonian University Medical College, 9 Medyczna Str., 30-688 Kraków, Poland*

[b] *IBMM, Université de Montpellier, CNRS, ENSCM, 34095 Montpellier, France*

[c] *Institut de Génomique Fonctionelle, Université de Montpellier, CNRS INSERM, 34094 Montpellier, France*

[d] *Maj Institute of Pharmacology, Polish Academy of Sciences, 12 Smętna Str., 31-343 Kraków, Poland*

* Corresponding author:
Paweł Zajdel
Department of Organic Chemistry
Jagiellonian University Medical College
E-mail: pawel.zajdel@uj.edu.pl
Tel.: +48 126205500

Table of contents

1. General procedures and characterization data for intermediates 1−4 ....S3

2. Characterization data for intermediates 5−6...S8

3. Characterization data for final compounds (except 18, 22, 27, 28 presented in the main manuscript)...S11

4. Spectra of selected final compound 27 ....S24

4.1 HRMS spectra S24

4.2 ^{1}H NMR and ^{13}C NMR spectra S25

5. The composition of the buffers used in radioligand binding assay...........................S26

6. *In vitro* safety space assessment for compound 27...S26

6.1 Evaluation of CYP450 inhibition..S26

6.2 Evaluation of mutagenic properties S27

7. References S28

1. General procedures and characterization data for intermediates 1−4

1.1 General procedure of aza-Baylis-Hillman reaction (1a–c)

In a dried flask p-toluenesulfonamide (1 eq) and 1,4-diazabicyclo[2.2.2]octane (DABCO) (0.15 eq) were mixed together with the previously activated molecular sieves (4A, 200 mg/mmol). The mixture was suspended in isopropanol, followed by addition of fluorine-substituted or unsubstituted benzaldehyde (1 eq) and methyl acrylate (1.1 eq). Subsequently, titanium isopropoxide ($Ti(iOPr)_4$) was added as a freshly prepared solution in isopropanol (0.02 eq). The flask was filled with nitrogen and the mixture was stirred at room temperature for 36 h. Then, a mixture was filtered through Celite which was rinsed with CH_2Cl_2. The solvent was evaporated and the remaining crude was dissolved in EtOAc, washed three times with 1 M $KHSO_4$, once with saturated $NaHCO_3$, water and brine and dried over Na_2SO_4. Evaporation of the solvent gave a yellow oil which was subsequently dissolved in EtOAc and precipitated upon portionwise addition of n-hexane. The appearing white precipitate was filtered, dried under vacuum and was pure enough to be directly engaged in the next step.

1.2 Characterization data for intermediates 1a–c

Methyl 2-(((4-methylphenyl)sulfonamido)(phenyl)methyl)acrylate (1a)

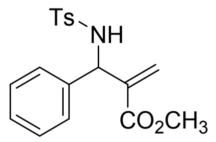

Figure S1. Chemical structure of compound **1a**

White solid, 16.8 g (yield 76%), UPLC/MS purity 100%, $t_R = 6.70$, $C_{18}H_{19}NO_4S$, MW 345.41, Monoisotopic Mass 345.10, $[M+H]^+$ 346.1. ^1H NMR (500 MHz, $CDCl_3$) δ ppm 2.41 (s, 3H), 3.61 (s, 3H), 5.31 (d, $J = 8.9$ Hz, 1H), 5.72 (d, $J = 8.9$ Hz, 1H), 5.84 (s, 1H), 6.23 (s, 1H), 7.13–7.17 (m, 2H), 7.20–7.26 (m, 5H), 7.66–7.70 (m, 2H). ^{13}C NMR (126 MHz, $CDCl_3$) δ ppm 21.6, 52.1, 59.1, 126.5, 127.3, 127.8, 128.0, 128.7, 129.6, 137.7, 138.6, 138.7, 143.5, 165.9.

Methyl 2-((3-fluorophenyl)((4-methylphenyl)sulfonamido)methyl)acrylate (1b)

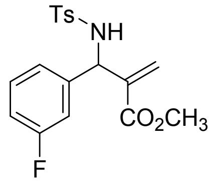

Figure S2. Chemical structure of compound **1b**

White solid, 32.4 g (yield 72%), UPLC/MS purity 100%, $t_R = 6.84$, $C_{18}H_{18}FNO_4S$, MW 363.40, Monoisotopic Mass 363.09, $[M+H]^+$ 364.1. ^1H NMR (500 MHz, $CDCl_3$) δ ppm 2.39 (s, 3H), 3.60 (s, 3H), 5.27 (d, $J = 9.2$ Hz, 1H), 5.78 (s, 1H), 5.80 (d, $J = 9.3$ Hz, 1H), 6.20 (s, 1H), 6.82–6.91 (m, 2H), 6.94 (dt, $J = 7.8$, 0.8 Hz, 1H), 7.15–7.26 (m, 3H), 7.64–7.67 (m, 2H). ^{13}C NMR (126 MHz, $CDCl_3$) δ ppm 21.6, 52.2, 58.8, 113.7 (d, $J = 22.9$ Hz), 114.8 (d, $J = 21.1$ Hz), 122.1 (d, $J = 2.4$ Hz), 127.3, 128.5, 129.6, 130.2 (d, $J = 8.5$ Hz), 137.6, 138.1, 141.4 (d, $J = 7.9$ Hz), 143.7, 162.9 (d, $J = 247.5$ Hz), 165.7.

Methyl 2-((4-fluorophenyl)((4-methylphenyl)sulfonamido)methyl)acrylate (1c)

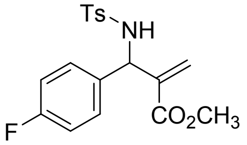

Figure S3. Chemical structure of compound **1c**

White solid, 7.3 g (yield 71%), UPLC/MS purity 100%, $t_R = 6.82$, $C_{18}H_{18}FNO_4S$, MW 363.40, Monoisotopic Mass 363.09, $[M+H]^+$ 364.2. ^1H NMR (500 MHz, $CDCl_3$) δ ppm 2.42 (s, 3H), 3.62 (s, 3H), 5.29 (d, $J = 8.9$ Hz, 1H), 5.76 (d, $J = 8.9$ Hz, 1H), 5.81 (s, 1H), 6.21 (s, 1H), 6.88–6.94 (m, 2H), 7.11–7.16 (m, 2H), 7.22–7.26 (m, 2H), 7.64–7.69 (m, 2H). ^{13}C NMR (126 MHz, $CDCl_3$) δ ppm 21.6, 52.2, 58.6, 115.5 (d, $J = 21.7$ Hz), 127.3, 128.1, 128.3 (d, $J = 7.9$ Hz), 129.6, 134.5 (d, $J = 3.0$ Hz), 137.6, 138.4, 143.6, 162.3 (d, $J = 246.3$ Hz), 165.77.

1.3 General procedure for *N*-allylation of β-Aminoester (2a–c)

β-Aminoester **1** (1 eq) was dissolved in DMF, followed by addition of K_2CO_3 (3 eq). Subsequently, allyl bromide (1.5 eq) was added dropwisely. The reaction was stirred at room temperature for 6 h. The mixture was diluted with ethyl acetate and washed 3 times with water and brine. The organic phase was filtered and evaporated. The remaining yellow residue was dissolved in EtOAc and precipitated upon portionwise addition of n-hexane. The obtained white solid was filtered and dried under vacuum.

1.4 Characterization data for intermediates 2a–c

Methyl 2-(((*N*-allyl-4-methylphenyl)sulfonamido)(phenyl)methyl)acrylate (2a)

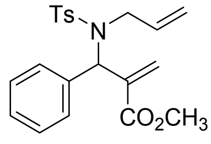

Figure S4. Chemical structure of compound **2a**

White solid, 6.40 g (yield 95%), UPLC/MS $t_R = 7.90$, $C_{21}H_{23}NO_4S$, MW 385.46, Monoisotopic Mass 385.13, $[M+H]^+$ 386.1. ^1H NMR (300 MHz, $CDCl_3$) δ ppm 2.43 (s, 3H), 3.60 (s, 3H), 3.76–3.85 (m, 2H), 4.78–4.87 (m, 2H), 5.19–5.35 (m, 1H), 5.73 (d, $J = 1.8$ Hz,

1H), 6.12 (s, 1H) 6.44 (s, 1H), 7.0 (dd, J = 6.5, 2.9 Hz, 2H), 7.19–7.29 (m, 5H), 7.69 (d, J = 8.2 Hz, 2H). ^{13}C NMR (75 MHz, $CDCl_3$) δ ppm 21.5, 48.7, 52.0, 61.8, 117.6, 127.5, 128.0, 128.4, 129.4, 134.3, 137.1, 137.9, 139.2, 143.1, 166.3.

Data in agreement with lit.[1,2]

Methyl 2-(((*N*-allyl-4-methylphenyl)sulfonamido)(3-fluorophenyl)methyl)acrylate (2b)

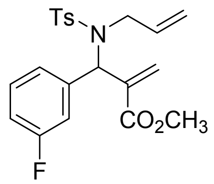

Figure S5. Chemical structure of compound **2b**

White solid, 14.8 g (yield 89%), UPLC/MS t_R = 8.01, $C_{21}H_{22}FNO_4S$, MW 403.47, Monoisotopic Mass 403.03, $[M+H]^+$ 404.2. ^{1}H NMR (500 MHz, $CDCl_3$) δ ppm 2.41 (s, 3H), 3.60 (s, 3H), 3.79–3.84 (m, 2H), 4.81–4.84 (m, 1H), 4.84–4.86 (m, 1H), 5.26 - 5.35 (m, 1H), 5.70 (d, J = 1.5 Hz, 1H), 6.07 (s, 1H), 6.44 (d, J = 1.1 Hz, 1H), 6.69 (dt, J = 9.8, 2.1 Hz, 1H), 6.83 (dt, J = 7.7, 0.8 Hz, 1H), 6.92 (tdd, J = 8.4, 8.4, 2.5, 0.8 Hz, 1H), 7.16–7.22 (m, 1H), 7.24–7.25 (m, 1H), 7.26 (d, J = 0.6 Hz, 1H), 7.65–7.68 (m, 2H). ^{13}C NMR (126 MHz, $CDCl_3$) δ ppm 21.6, 49.0, 52.2, 61.3, 115.1 (d, J = 21.1 Hz), 115.7 (d, J = 21.7 Hz), 117.9, 124.3 (d, J = 2.4 Hz), 127.6, 128.8, 129.6, 130.1 (d, J = 8.5 Hz), 134.2, 137.8, 138.7, 140.0 (d, J = 6.6 Hz) 143.5, 162.8 (d, J = 245.7 Hz), 166.2.

Methyl 2-(((*N*-allyl-4-methylphenyl)sulfonamido)(4-fluorophenyl)methyl)acrylate (2c)

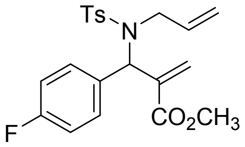

Figure S6. Chemical structure of compound **2c**

White solid, 10.3 g (yield 93%), UPLC/MS t_R = 8.03, $C_{21}H_{22}FNO_4S$, MW 403.47, Monoisotopic Mass 403.03, $[M+H]^+$ 404.2. ^{1}H NMR (500 MHz, $CDCl_3$) δ ppm 2.42 (s, 3H), 3.59 (s, 3H), 3.76 (ddt, J = 16.0, 5.7, 1.4, 1.4 Hz, 1H), 3.80–3.87 (m, 1H), 4.79–4.83 (m, 1H), 4.83–4.85 (m, 1H), 5.23–5.33 (m, 1H), 5.70 (d, J = 1.43 Hz, 1H), 6.06 (s, 1H), 6.42 (d, J = 1.43 Hz, 1H), 6.88–6.94 (m, 2H), 6.97–7.02 (m, 2H), 7.23–7.28 (m, 2H), 7.63–7.68 (m, 2H). ^{13}C NMR (126 MHz, $CDCl_3$) δ ppm 21.6, 48.9, 52.2, 61.2, 115.6 (d, J = 21.1 Hz), 117.8, 127.5, 128.2, 129.6, 130.4 (d, J = 7.9 Hz), 133.10 (d, J = 3.0 Hz), 134.3, 137.9, 139.0, 143.4, 162.5 (d, J = 248.1 Hz), 166.3.

1.5 General procedure for ring-closing metathesis (3a–c)

Diene compound **2** (1 eq) was dissolved in EtOAc and NO_2-Grela catalyst (1 mol %) was added in three portions over a period of 1.5 h with 30 min time intervals. The reaction was quenched with ethyl vinyl ether (50 eq/Ru) and stirred for additional 1 h. The solution was evaporated and the remaining residue was dissolved in CH_2Cl_2 and filtered through a

layer of silica gel. The filtrate was evaporated and the obtained residue was treated with diethyl ether giving a grey-white precipitate, which was filtrated off and dried under reduced pressure.

1.6 Characterization data for intermediates 3a–c

Methyl 2-phenyl-1-tosyl-2,5-dihydro-1*H*-pyrrole-3-carboxylate (3a)

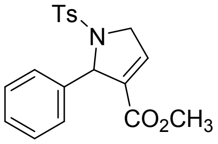

Figure S7. Chemical structure of compound **3a**

White solid, 1.66 g (yield 58%), UPLC/MS[1] $t_R = 7.05$, $C_{19}H_{19}NO_4S$, MW 357.42, Monoisotopic Mass 357.1, $[M+H]^+$ 358.1. ^1H NMR (300 MHz, $CDCl_3$) δ ppm 2.37 (s, 3H), 3.58 (s, 3H), 4.34–4.43 (m, 1H), 4.47–4.57 (m, 1H), 5.73–5.76 (m, 1H), 6.76–6.80 (m, 1H), 7.14 (d, $J = 8.1$ Hz, 2H), 7.20–7.25 (m, 5H), 7.39–7.45 (m, 2H). ^{13}C NMR (75 MHz, $CDCl_3$) δ ppm 21.6, 51.8, 55.0, 69.0, 127.1, 127.7, 128.3, 128.5, 129.5, 135.6, 135.7, 139.4, 143.3, 162.2.

Data in agreement with lit.[1,3]

Methyl 2-(3-fluorophenyl)-1-tosyl-2,5-dihydro-1*H*-pyrrole-3-carboxylate (3b)

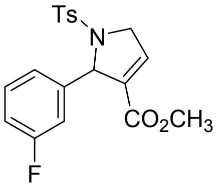

Figure S8. Chemical structure of compound **3b**

White solid, 4.09 g (yield 61%), UPLC/MS[1] $t_R = 7.10$, $C_{19}H_{18}FNO_4S$, MW 375.41, Monoisotopic Mass 375.09, $[M+H]^+$ 376.2. ^1H NMR (500 MHz, $CDCl_3$) δ ppm 2.39 (s, 3H), 3.61 (s, 3H), 4.41 (ddd, $J = 17.3$, 5.7, 2.0 Hz, 1H), 4.52 (dt, $J = 17.3$, 2.6 Hz, 1H), 5.70–5.73 (m, 1H), 6.80 (q, $J = 2.1$ Hz, 1H), 6.88 (dt, $J = 9.7$, 2.1 Hz, 1H), 6.94 (tdd, $J = 8.4$, 8.4, 2.6, 0.9 Hz, 1H), 7.08 (dt, $J = 7.7$, 1.2 Hz, 1H), 7.17–7.20 (m, 2H), 7.20–7.26 (m, 1H), 7.45–7.49 (m, 2H). ^{13}C NMR (126 MHz, $CDCl_3$) δ ppm 21.6, 52.0, 55.1, 68.5, 114.6 (d, $J = 22.3$ Hz), 115.1 (d, $J = 21.7$ Hz), 123.7 (d, $J = 2.4$ Hz), 127.2, 129.7, 129.9 (d, $J = 8.5$ Hz), 135.4, 135.4, 136.2, 142.1 (d, $J = 6.6$ Hz), 143.7, 162.1, 162.8 (d, $J = 246.3$ Hz).

Methyl 2-(4-fluorophenyl)-1-tosyl-2,5-dihydro-1*H*-pyrrole-3-carboxylate (3c)

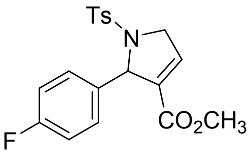

Figure S9. Chemical structure of compound **3c**

White solid, 3.25 g (yield 62%), UPLC/MS[1] $t_R = 7.10$, $C_{19}H_{18}FNO_4S$, MW 375.41, Monoisotopic Mass 375.09, $[M+H]^+$ 376.2. ^1H NMR (500 MHz, $CDCl_3$) δ ppm 2.38 (s, 3H), 3.61 (s, 3H), 4.33–4.43 (m, 1H), 4.45–4.55 (m, 1H), 5.68–5.74 (m, 1H), 6.80 (q, $J = 2.1$ Hz, 1H), 6.88–6.93 (m, 2H), 6.96–7.02 (m, 2H), 7.23–7.28 (m, 2H), 7.64–7.68 (m, 2H). ^{13}C NMR (126 MHz, $CDCl_3$) δ ppm 21.63, 48.9, 52.2, 61.2, 115.6 (d, $J = 21.7$ Hz), 127.5, 129.6, 130.5 (d, $J = 8.5$ Hz), 133.1 (d, $J = 3.0$ Hz), 134.3, 137.8, 139.0, 143.4, 162.5 (d, $J = 249.9$ Hz), 166.3.

1.7 General procedure for tosyl group deprotection and aromatization (4a–c)

To a solution of compound **3** (1 eq) in DMF, sodium *tert*-butoxide (3 eq) was added portion-wise. The reaction was carried for 2 h under TLC monitoring. The mixture was diluted with EtOAc, neutralized with 1 M $KHSO_4$, washed with saturated solution of $NaHCO_3$, water and brine. The organic layer was dried with Na_2SO_4 and evaporated. The obtained crude product was purified on silica gel with EtOAc/Hex (4/6 *v/v*) as a developing solvent.

1.8 Characterization data for intermediates 4a–c

Methyl 2-phenyl-1*H*-pyrrole-3-carboxylate (4a)

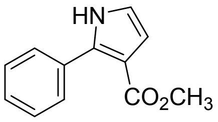

Figure S10. Chemical structure of compound **4a**

White oil, 1.38 g (yield 62%) after chromatographic purification over silica gel with EtOAc/Hex (4/6, *v/v*), UPLC/MS purity 100%, $t_R = 5.36$, $C_{12}H_{11}NO_2$, MW 201.23, Monoisotopic Mass 201.08, $[M+H]^+$ 202.1. ^1H NMR (300 MHz, $CDCl_3$) δ ppm 3.71 (s, 3H), 6.66–6.75 (m, 2H), 7.31–7.36 (m, 2H), 7.38–7.41 (m, 1H), 7.52–7.55 (m, 1H), 7.55–7.58 (m, 1H), 8.68 (s, 1H). ^{13}C NMR (75 MHz, $CDCl_3$) δ ppm 51.0, 111.7, 112.1, 117.9, 128.1, 128.9, 132.0, 137.3, 165.6.

Data in agreement with lit.[1,4]

Methyl 2-(3-fluorophenyl)-1*H*-pyrrole-3-carboxylate (4b)

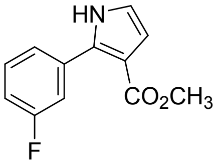

Figure S11. Chemical structure of compound **4b**

Yellow solid, 1.38 g (yield 79%) after chromatographic purification over silica gel with EtOAc/Hex (4/6, *v/v*), UPLC/MS purity 100%, $t_R = 5.83$, $C_{12}H_{10}FNO_2$, MW 219.21, Monoisotopic Mass 219.07, $[M+H]^+$ 220.2. ^{1}H NMR (500 MHz, $CDCl_3$) δ ppm 3.76 (s, 3H), 6.75 (dt, *J* = 12.7, 2.8 Hz, 2H), 7.02–7.08 (m, 1H), 7.30–7.37 (m, 3H), 8.64 (br. s., 1H). ^{13}C NMR (126 MHz, $CDCl_3$) δ ppm 51.20, 112.4, 112.5, 115.2 (d, *J* = 21.7 Hz), 116.1 (d, *J* = 22.9 Hz), 118.3, 124.6 (d, *J* = 2.4 Hz), 129.8 (d, *J*=8.5 Hz), 134.1 (d, *J* = 9.1 Hz), 135.7, 162.5 (d, *J* = 246.3 Hz), 165.4.

Methyl 2-(4-fluorophenyl)-1*H*-pyrrole-3-carboxylate (4c)

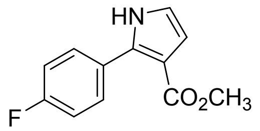

Figure S12. Chemical structure of compound **4c**

Yellow solid, 1.3 g (yield 74%) after chromatographic purification over silica gel with EtOAc/Hex (4/6, *v/v*), UPLC/MS purity 99%, $t_R = 5.85$, $C_{12}H_{10}FNO_2$, MW 219.21, Monoisotopic Mass 219.07, [M+H]+ 220.2. ^{1}H NMR (500 MHz, $CDCl_3$) δ ppm 3.73 (s, 3H), 6.70–6.74 (m, 2H), 7.04–7.10 (m, 2H), 7.50–7.56 (m, 2H), 8.66 (br. s., 1H). ^{13}C NMR (126 MHz, $CDCl_3$) δ ppm 51.1, 111.9, 112.2, 115.2 (d, *J* = 21.7 Hz), 117.9, 128.2 (d, *J* = 3.6 Hz), 130.8 (d, *J* = 9.1 Hz), 136.35, 163.73 (d, *J* = 249.9 Hz), 165.6.

2. Characterization data for intermediates 5−6

2.1 Characterization data for intermediates 5a–c

2-phenyl-1*H*-pyrrole-3-carboxylic acid (5a)

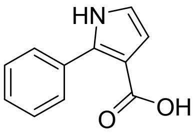

Figure S13. Chemical structure of compound **5a**

Brown solid, 2.6 g (yield 88%), UPLC/MS $t_R = 3.62$, $C_{11}H_9NO_2$, MW 187.19, Monoisotopic Mass 187.06, $[M+H]^+$ 188.0. ^{1}H NMR (500 MHz, CD_3OD) δ ppm 6.62 (d, *J* = 3.1 Hz, 1H), 6.74 (d, *J* = 2.9 Hz, 1H), 7.26–7.30 (m, 1H), 7.32–7.36 (m, 2H), 7.54–7.58 (m, 2H). ^{13}C NMR (126 MHz, CD_3OD) δ ppm 111.2, 111.8, 117.7, 127.4, 127.6, 128.9, 132.7, 137.5, 167.9.

2-(3-Fluorophenyl)-1*H*-pyrrole-3-carboxylic acid (5b)

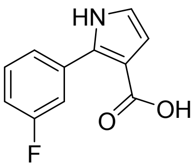

Figure S14. Chemical structure of compound **5b**

Brown solid, 1.05 (yield 74%), UPLC/MS purity 100%, $t_R = 3.94$, $C_{11}H_8FNO_2$, MW 205.19, Monoisotopic Mass 205.05, $[M+H]^+$ 206.2. ^{1}H NMR (500 MHz, CD_3OD) δ ppm 6.64 (d,

J = 3.0 Hz, 1H), 6.76 (d, J = 2.9 Hz, 1H), 6.99–7.04 (m, 1H), 7.31–7.39 (m, 3H). ^{13}C NMR (126 MHz, CD_3OD) δ ppm 111.8, 112.1, 114.0 (d, J = 21.1 Hz), 115.7 (d, J = 22.3 Hz), 118.1, 124.5 (d, J = 3.0 Hz), 129.3 (d, J = 8.5 Hz), 134.8 (d, J = 8.5 Hz), 135.7, 162.4 (d, J = 243.9 Hz), 167.6.

2-(4-Fluorophenyl)-1*H*-pyrrole-3-carboxylic acid (5c)

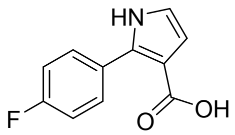

Figure S15. Chemical structure of compound **5c**

Brown solid, 1.60 (yield 82%), UPLC/MS purity 100%, t_R = 3.89, $C_{11}H_8FNO_2$, MW 205.19, Monoisotopic Mass 205.05, [M+H]+ 206.1. ^{1}H NMR (500 MHz, CD_3OD) δ ppm 6.61 (d, J = 2.9 Hz, 1H), 6.73 (d, J = 2.9 Hz, 1H), 7.04–7.10 (m, 2H), 7.54–7.60 (m, 2H). ^{13}C NMR (126 MHz, CD_3OD) δ ppm 111.3, 111.8, 114.3 (d, J = 21.7 Hz), 117.7, 128.9 (d, J = 3.0 Hz), 130.9 (d, J = 8.5 Hz), 136.4, 162.5 (d, J = 245.7 Hz), 167.7.

2.2 Characterization data for intermediates 6a–f

***Tert*-butyl (*R*)-3-(2-phenyl-1*H*-pyrrole-3-carboxamido)-pyrrolidine-1-carboxylate (6a)**

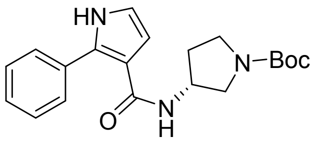

Figure S16. Chemical structure of compound **6a**

White solid, 1 g (yield 75%) after chromatographic purification over silica gel with EtOAc/Hex (8/2, *v/v*); UPLC/MS purity 99%, t_R = 5.61, $C_{20}H_{25}N_3O_3$, MW 355.44, Monoisotopic Mass 355.19, $[M+H]^+$ 356.2. ^{1}H NMR (400 MHz, CD_3OD) δ ppm 1.44 (s, 9H), 1.75–1.88 (m, 1H), 2.02–2.16 (m, 1H), 3.12–3.22 (m, 1H), 3.23–3.37 (m, 2H), 3.51–3.59 (m, 1H), 4.36–4.46 (m, 1H), 6.49 (d, J = 2.2 Hz, 1H), 6.76 (d, J = 2.7 Hz, 1H), 7.26–7.33 (m, 1H), 7.37 (t, J = 7.4 Hz, 2H), 7.49 (d, J = 7.4 Hz, 2H). ^{13}C NMR (100 MHz, CD_3OD) δ ppm 27.5, 29.8, 30.7, 43.6, 44.1, 48.8, 49.6, 50.4, 51.0, 109.5, 117.9, 127.3, 128.2, 132.6, 168.0

***Tert*-butyl (*S*)-3-(2-phenyl-1*H*-pyrrole-3-carboxamido)-pyrrolidine-1-carboxylate (6b)**

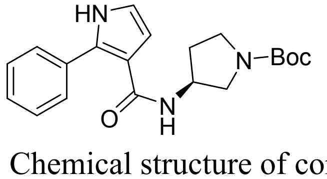

Figure S17. Chemical structure of compound **6b**

White solid, 0.49 g (yield 86%), after chromatographic purification over silica gel with EtOAc/Hex (8/2, *v/v*); UPLC/MS purity 96%, t_R = 5.59, $C_{20}H_{25}N_3O_3$, MW 355.44, Monoisotopic Mass 355.19, $[M+H]^+$ 356.2. ^{1}H NMR (400 MHz, CD_3OD) δ ppm 1.44 (s, 9H), 1.74–1.87 (m, 1H), 2.02–2.17 (m, 1H), 3.12–3.22 (m, 1H), 3.22–3.37 (m, 2H), 3.50–3.59 (m,

1H), 4.36–4.46 (m, 1H), 6.49 (d, *J* = 2.2 Hz, 1H), 6.76 (d, *J* = 2.7 Hz, 1H), 7.25–7.33 (m, 1H), 7.37 (t, *J* = 7.4 Hz, 2H), 7.49 (d, *J* = 7.4 Hz, 2H). ^{13}C NMR (100 MHz, CD_3OD) δ ppm 27.5, 29.9, 30.8, 43.6, 44.1, 48.8, 49.6, 50.4, 51.0, 109.5, 117.9, 127.4, 128.2, 132.6, 168.0.

***Tert*-butyl (*R*)-3-[2-(3-fluorophenyl)-1*H*-pyrrole-3-carboxamido]-pyrrolidine-1-carboxylate (6c)**

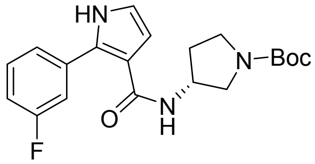

Figure S18. Chemical structure of compound **6c**

Yellow oil, 0.19 g (yield 52%) after chromatographic purification over silica gel with EtOAc/Hex (8/2, *v/v*); UPLC/MS purity 99%, t_R = 5.76, $C_{20}H_{24}FN_3O_3$, MW 373.43, Monoisotopic Mass 373.18, $[M+H]^+$ 374.2. ^{1}H NMR (400 MHz, CD_3OD) δ ppm 1.44 (s, 9H), 1.81–1.94 (m, 1H), 2.05–2.19 (m, 1H), 3.21 (dd, *J* = 10.8, 4.7 Hz, 1H), 3.31–3.41 (m, 2H), 3.54–3.64 (m, 1H), 4.44 (quin, *J* = 5.9 Hz, 1H), 6.49 (d, *J* = 2.9 Hz, 1H), 6.78 (d, *J* = 2.9 Hz, 1H), 6.96–7.05 (m, 1H), 7.26–7.40 (m, 3H). ^{13}C NMR (100 MHz, CD_3OD) δ ppm 28.9, 31.3, 32.2, 45.1, 45.6, 50.3, 51.0, 51.8, 52.4, 111.1, 115.1 (d, *J* = 20.5 Hz), 116.1 (d, *J* = 22.0 Hz), 117.5, 119.8, 125.2, 131.2 (d, *J* = 8.8 Hz), 133.4 (d, *J* = 2.9 Hz), 136.3 (d, *J* = 7.3 Hz), 164.2 (d, *J* = 243.6 Hz), 169.4.

***Tert*-butyl (*S*)-3-[2-(3-fluorophenyl)-1*H*-pyrrole-3-carboxamido]-pyrrolidine-1-carboxylate (6d)**

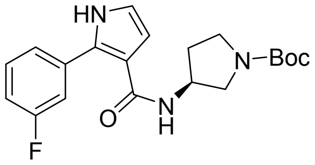

Figure S19. Chemical structure of compound **6d**

Yellow oil, 0.25 g (yield 68%) after chromatographic purification over silica gel with EtOAc/Hex (8/2, *v/v*); UPLC/MS purity 98%, t_R = 5.77, $C_{20}H_{24}FN_3O_3$, MW 373.43, Monoisotopic Mass 373.18, $[M+H]^+$ 374.2. ^{1}H NMR (400 MHz, CD_3OD) δ ppm 1.44 (s, 9H), 1.80–1.94 (m, 1H), 2.06–2.19 (m, 1H), 3.21 (dd, *J* = 10.8, 4.7 Hz, 1H), 3.31–3.40 (m, 2H), 3.54–3.64 (m, 1H), 4.44 (quin, *J* = 5.9 Hz, 1H), 6.49 (d, *J* = 2.9 Hz, 1H), 6.78 (d, *J* = 2.9 Hz, 1H), 6.96–7.05 (m, 1H), 7.26–7.40 (m, 3H). ^{13}C NMR (100 MHz, CD_3OD) δ ppm 28.9, 31.3, 32.2, 45.1, 45.6, 50.3, 51.0, 51.8, 52.4, 111.1, 115.1 (d, *J* = 20.5 Hz), 116.1 (d, *J* = 22.0 Hz), 117.5, 119.8, 125.2, 131.2 (d, *J* = 8.8 Hz), 133.4 (d, *J* = 2.9 Hz), 136.3 (d, *J* = 7.3 Hz), 164.2 (d, *J* = 243.6 Hz), 169.4.

***Tert*-butyl (*R*)-3-[2-(4-fluorophenyl)-1*H*-pyrrole-3-carboxamido]-pyrrolidine-1-carboxylate (6e)**

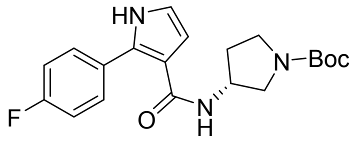

Figure S20. Chemical structure of compound **6e**

White solid, 0.60 g (yield 82%), after chromatographic purification over silica gel with EtOAc/Hex (8/2, *v/v*); UPLC/MS purity 100%, $t_R = 5.77$, $C_{20}H_{24}FN_3O_3$, MW 373.43, Monoisotopic Mass 373.18, $[M+H]^+$ 374.2. ^{1}H NMR (400 MHz, CD_3OD) δ ppm 1.45 (s, 9H), 1.78–1.92 (m, 1H), 2.06–2.18 (m, 1H), 3.19 (dd, *J* = 11.2, 5.1 Hz, 1H), 3.28–3.36 (m, 2H), 3.51–3.61 (m, 1H), 4.41 (quin, *J* = 5.5 Hz, 1H), 6.53 (d, *J* = 2.5 Hz, 1H), 6.75 (d, *J* = 3.1 Hz, 1H), 7.06–7.13 (m, 2H), 7.51 (dd, *J* = 8.7, 5.4 Hz, 2H). ^{13}C NMR (100 MHz, CD_3OD) δ ppm 28.9, 31.4, 32.2, 45.1, 45.6, 50.3, 51.0, 51.9, 52.4, 61.7, 81.1, 110.8, 116.2 (d, *J* = 22.0 Hz), 119.3, 130.4 (d, *J* = 2.9 Hz), 131.6 (d, *J* = 7.3 Hz), 163.8 (d, *J* = 245.0 Hz), 169.3.

***Tert*-butyl (*S*)-3-[2-(4-fluorophenyl)-1*H*-pyrrole-3-carboxamido]-pyrrolidine-1-carboxylate (6f)**

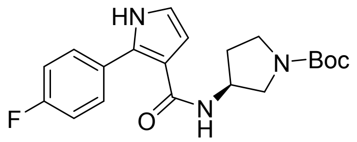

Figure S21. Chemical structure of compound **6f**

White solid, 0.55 g (yield 76%) after chromatographic purification over silica gel with EtOAc/Hex (8/2, *v/v*); UPLC/MS purity 100%, $t_R = 5.77$, $C_{20}H_{24}FN_3O_3$, MW 373.43, Monoisotopic Mass 373.18, $[M+H]^+$ 374.2. ^{1}H NMR (400 MHz, CD_3OD) δ ppm 1.45 (s, 9H), 1.79–1.92 (m, 1H), 2.05–2.17 (m, 1H), 3.19 (dd, *J* = 11.2, 5.1 Hz, 1H), 3.28–3.36 (m, 2H), 3.52–3.61 (m, 1H), 4.41 (quin, *J* = 5.5 Hz, 1H), 6.53 (d, *J* = 2.5 Hz, 1H), 6.75 (d, *J* = 3.1 Hz, 1H), 7.06–7.13 (m, 2H), 7.51 (dd, *J* = 8.7, 5.4 Hz, 2H). ^{13}C NMR (100 MHz, CD_3OD) δ ppm 28.9, 31.4, 32.2, 45.1, 45.6, 50.3, 51.0, 51.9, 52.4, 61.7, 81.1, 110.8, 116.2 (d, *J* = 22.0 Hz), 119.3, 130.4 (d, *J* = 2.9 Hz), 131.6 (d, *J* = 7.3 Hz), 163.8 (d, *J* = 245.0 Hz), 169.3.

3. Characterization data for final compounds (except 18, 22, 27, 28 presented in the main manuscript)

(*R*)-2-Phenyl-1-(phenylsulfonyl)-*N*-(pyrrolidin-3-yl)-1*H*-pyrrole-3-carboxamide (7)

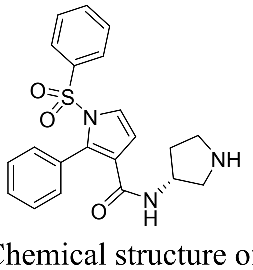

Figure S22. Chemical structure of compound **7**

Boc derivative: colorless oil, 0.15 g (yield 70%) after chromatographic purification over silica gel with EtOAc/Hex (6/4, *v/v*); UPLC/MS purity 99%, $t_R = 7.75$, $C_{26}H_{29}N_3O_5S$, MW 495.59, Monoisotopic Mass 495.18, $[M+H]^+$ 496.2.

Hydrochoride: white solid, 0.10 g (yield 76%), UPLC/MS purity 100%, $t_R = 4.62$, $C_{21}H_{22}ClN_3O_3S$, MW 431.94. ^{1}H NMR: (300 MHz, CD_3OD) δ ppm 1.70–1.80 (m, 1H), 2.10–2.20 (m, 1H), 3.01 (dd, J = 12.3, 5.1 Hz, 1H), 3.17–3.27 (m, 2H), 3.37 (dd, J = 12.3, 7.1 Hz, 1H), 4.23–4.32 (m, 1H), 6.73 (d, J = 3.5 Hz, 1H), 7.00–7.06 (m, 2H), 7.25–7.35 (m, 4H), 7.41 (t, J = 7.6 Hz, 3H), 7.59 (d, J = 3.5 Hz, 1H), 7.61–7.68 (m, 1H). ^{13}C NMR (75 MHz, CD_3OD) δ ppm 30.8, 45.6, 50.2, 50.8, 111.4, 123.8, 124.0, 127.1, 128.5, 128.6, 130.0, 130.3, 130.4, 130.6, 133.1, 135.6, 136.7, 139.4, 166.7. Monoisotopic Mass for free base $C_{21}H_{21}N_3O_3S$: 395.13, $[M+H]^+$ 396.1.

(*S*)-2-Phenyl-1-(phenylsulfonyl)-*N*-(pyrrolidin-3-yl)-1*H*-pyrrole-3-carboxamide (8)

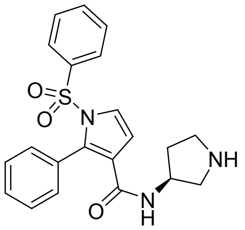

Figure S23. Chemical structure of compound **8**

Boc-derivative: colorless oil, 0.08 g (yield 57%) after chromatographic purification over silica gel with EtOAc/Hex (6/4, *v/v*); UPLC/MS purity 100%, $t_R = 7.30$, $C_{26}H_{29}N_3O_5S$, MW 495.59, Monoisotopic Mass 495.18, $[M+H]^+$ 496.1.

Hydrochloride: white solid, 0.06 g (yield 77%), UPLC/MS purity 100%, $t_R = 4.24$, $C_{21}H_{22}ClN_3O_3S$, MW 431.94. ^{1}H NMR: (300 MHz, CD_3OD) δ ppm 1.70–1.81 (m, 1H), 2.11–2.22 (m, 1H), 3.01 (dd, J = 12.3, 5.3 Hz, 1H), 3.17–3.27 (m, 2H), 3.37 (dd, J = 12.3, 7.0 Hz, 1H), 4.23–4.32 (m, 1H), 6.73 (d, J = 3.5 Hz, 1H), 7.00–7.06 (m, 2H), 7.27–7.35 (m, 4H), 7.41 (t, J = 7.6 Hz, 3H), 7.59 (d, J = 3.5 Hz, 1H), 7.60–7.67 (m, 1H). ^{13}C NMR (75 MHz, CD_3OD) δ ppm 30.8, 45.6, 50.2, 50.8, 111.4, 123.8, 124.0, 127.1, 128.5, 128.6, 130.0, 130.3, 130.4, 130.6, 133.1, 135.6, 136.7, 139.4, 166.7. Monoisotopic Mass for free base $C_{21}H_{21}N_3O_3S$: 395.13, $[M+H]^+$ 396.1.

(*R*)-2-Phenyl-1-[(3-methylphenyl)sulfonyl]-*N*-(pyrrolidin-3-yl)-1*H*-pyrrole-3-carboxamide (9)

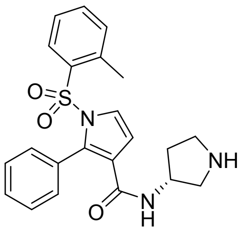

Figure S24. Chemical structure of compound **9**

Boc-derivative: colorless oil, 0.17 g (yield 70%) after chromatographic purification over silica gel with EtOAc/Hex (6/4, v/v); UPLC/MS purity 97%, t_R = 7.66, $C_{27}H_{31}N_3O_5S$, MW 509.62, Monoisotopic Mass 509.20, $[M+H]^+$ 510.2.

Hydrochloride: white solid, 0.11 g (yield 74%); UPLC/MS purity 100%, t_R = 4.56, $C_{22}H_{24}ClN_3O_3S$, Molecular Weight 445.96. ^{1}H NMR: (500 MHz, CD_3OD) δ ppm 1.71–1.80 (m, 1H), 2.10–2.19 (m, 1H), 2.24 (s, 3H), 3.04 (dd, *J* = 12.2, 4.7 Hz, 1H), 3.18–3.28 (m, 2H), 3.36 (dd, *J* = 12.0, 7.2 Hz, 1H), 4.26–4.33 (m, 1H), 6.78 (d, *J* = 3.4 Hz, 1H), 6.90 (d, *J* = 6.9 Hz, 2H), 6.92–6.97 (m, 1H), 7.02–7.05 (m, 1H), 7.13 (t, *J* = 7.9 Hz, 2H), 7.25–7.32 (m, 2H), 7.43 (td, *J* = 7.5, 1.4 Hz, 1H), 7.63 (d, *J* = 3.7 Hz, 1H). ^{13}C NMR (126 MHz, CD_3OD) δ ppm 18.2. 29.5. 44.3, 47.7, 48.8, 49.5, 108.9, 121.9, 122.5, 126.1, 127.3, 128.8, 128.9, 129.6, 129.9, 131.5, 131.8, 132.3, 134.3, 135.2, 136.1, 137.8, 165.1. Monoisotopic Mass for free base $C_{22}H_{23}N_3O_3S$: 409.15, $[M+H]^+$ 410.1.

(*R*)-2-Phenyl-1-[(2-chlorophenyl)sulfonyl]-*N*-(pyrrolidin-3-yl)-1*H*-pyrrole-3-carboxamide (10)

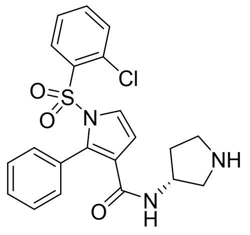

Figure S25. Chemical structure of compound **10**

Boc-derivative: colorless oil, 0.16 g (yield 86%) after chromatographic purification over silica gel with CH_2Cl_2/MeOH (9/0.2, *v/v*); UPLC/MS purity 99%, t_R = 7.71, $C_{26}H_{28}ClN_3O_5S$, MW 530.04, Monoisotopic Mass 529.14, $[M+H]^+$ 530.1.

Hydrochloride: white solid, 0.09 g (yield 64%); UPLC/MS purity 100%, t_R = 4.48, $C_{21}H_{21}Cl_2N_3O_3S$, MW 466.38. ^{1}H NMR (500 MHz, CD_3OD) δ ppm 1.76–1.84 (m, 1H), 2.12–2.21 (m, 1H), 3.07 (dd, *J* = 12.3, 4.9 Hz, 1H), 3.19–3.29 (m, 2H), 3.38 (dd, *J* = 12.0, 7.2 Hz, 1H), 4.27–4.35 (m, 1H),, 6.78 (d, *J* = 3.4 Hz, 1H), 6.94 (dd, *J* = 8.3, 1.2 Hz, 2H), 7.07–7.11 (m, 1H), 7.13 (t, *J* = 7.9 Hz, 2H), 7.19 (dd, *J* = 8.0, 1.2 Hz, 1H), 7.27–7.32 (m, 1H), 7.50–7.57 (m, 2H), 7.64 (d, *J* = 3.7 Hz, 1H). ^{13}C NMR (126 MHz, CD_3OD) δ ppm 29.5, 44.3, 48.9, 49.5, 108.8, 122.0, 123.3, 127.1. 127.4, 128.9, 128.9, 131.5, 131.7, 131.9, 132.2, 135.1, 135.2, 135.6, 165.0. Monoisotopic Mass for free base $C_{21}H_{20}ClN_3O_3S$: 429.09, $[M+H]^+$ 430.1.

(*R*)-2-Phenyl-1-[(3-methylphenyl)sulfonyl]-*N*-(pyrrolidin-3-yl)-1*H*-pyrrole-3-carboxamide (11)

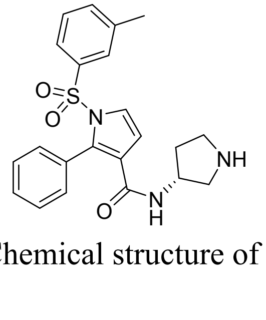

Figure S26. Chemical structure of compound **11**

Boc-derivative: colorless oil, 0.13 g (yield 73%) after chromatographic purification over silica gel with CH_2Cl_2/MeOH (9/0.3, v/v); UPLC/MS purity 98%, $t_R = 7.69$, $C_{27}H_{31}N_3O_5S$, MW 509.62, Monoisotopic Mass 509.20, $[M+H]^+$ 510.2.

Hydrochloride: white solid, 0.08 g (yield 73%); UPLC/MS purity 100%, $t_R = 4.66$, $C_{22}H_{24}ClN_3O_3S$, MW 445.96. ^1H NMR (500 MHz, CD_3OD) δ ppm 1.71–1.80 (m, 1H), 2.10–2.19 (m, 1H), 2.25 (s, 3H), 3.02 (dd, *J* = 12.0, 4.9 Hz, 1H), 3.17–3.28 (m, 2H), 3.37 (dd, *J* = 12.2, 7.0 Hz, 1H), 4.26–4.33 (m, 1H), 6.74 (d, *J* = 3.4 Hz, 1H), 6.99–7.04 (m, 3H), 7.16–7.20 (m, 1H), 7.30 (td, *J* = 7.8, 4.7 Hz, 3H), 7.41–7.46 (m, 2H), 7.56 (d, *J* = 3.4 Hz, 1H). ^{13}C NMR (126 MHz, CD_3OD) δ ppm 19.9, 29.5, 44.3, 48.8, 49.5, 110.0, 122.3, 122.5, 124.3, 127.2, 127.8, 128.9, 129.0, 129.3, 131.9, 135.0, 135.4, 137.7, 139.6, 165.3. Monoisotopic Mass for free base $C_{22}H_{23}N_3O_3S$: 409.15, $[M+H]^+$ 410.1.

(*R*)-2-Phenyl-1-[(3-fluorophenyl)sulfonyl]-*N*-(pyrrolidin-3-yl)-1*H*-pyrrole-3-carboxamide (12)

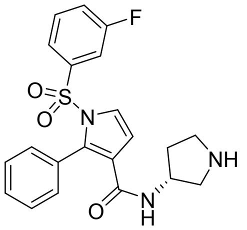

Figure S27. Chemical structure of compound **12**

Boc derivative: colorless oil, 0.16 g (yield 75%) after chromatographic purification over silica gel with EtOAc/Hex (6/4, v/v); UPLC/MS purity 100%, $t_R = 7.47$, $C_{26}H_{28}FN_3O_5S$, MW 513.58, Monoisotopic Mass 513.17, $[M+H]^+$ 514.3.

Hydrochloride: white solid, 0.10 g (yield 71%); UPLC/MS purity 100%, $t_R = 4.44$, $C_{21}H_{21}ClFN_3O_3S$, MW 449.93. ^1H NMR (300 MHz, CD_3OD) δ ppm 1.72–1.82 (m, 1H), 2.10–2.22 (m, 1H), 3.02 (dd, *J* = 12.3, 5.0 Hz, 1H), 3.17–3.29 (m, 2H), 3.88 (dd, *J* = 12.3, 7.0 Hz, 1H), 4.28 (pent, *J* = 7.0 Hz,1H), 6.73 (d, *J* = 3.5 Hz, 1H), 6.98 (td, *J* = 7.8, 2.1 Hz, 1H), 7.02–7.10 (m, 1H), 7.22 (dt, *J* = 7.6, 1.4 Hz, 1H), 7.31–7.35 (m, 2H), 7.38–7.50 (m, 4H), 7.60 (d, *J* = 3.7 Hz, 1H). ^{13}C NMR (75 MHz, CD_3OD) δ ppm 30.7, 45.6, 50.2, 50.8, 111.7, 115.8 (d, *J* = 26.4 Hz), 122.7, 122.8, 123.9, 124.2, 124.6, 128.7, 130.4 (d, *J* = 10.4 Hz), 132.7 (d, *J* = 8.0 Hz), 133.1, 136.8, 141.1 (d, *J* = 4.8 Hz), 163.4 (d, *J* = 251.7 Hz), 166.5. Monoisotopic Mass for free base $C_{21}H_{20}FN_3O_3S$: 413.12, $[M+H]^+$ 414.2.

(*R*)-2-Phenyl-1-[(3-trifluoromethylphenyl)sulfonyl]-*N*-(pyrrolidin-3-yl)-1*H*-pyrrole-3-carboxamide (13)

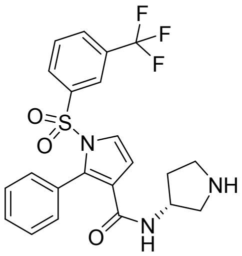

Figure S28. Chemical structure of compound **13**

Boc-derivative: colorless oil, 0.14 g (yield 71%) after chromatographic purification over silica gel with CH_2Cl_2/MeOH (9/0.2, v/v); UPLC/MS purity 98%, t_R = 8.02, $C_{27}H_{28}F_3N_3O_5S$, MW 563.59, Monoisotopic Mass 563.17, $[M+H]^+$ 564.2.

Hydrochloride: white solid, 0.08 g (yield 68%); UPLC/MS purity 100%, t_R = 5.05, $C_{22}H_{21}ClF_3N_3O_3S$, MW 499.93. ^{1}H NMR (500 MHz, CD_3OD) δ ppm 1.72–1.81 (m, 1H), 2.10–2.20 (m, 1H), 3.02 (dd, *J* = 12.0, 4.9 Hz, 1H), 3.17–3.28 (m, 2H), 3.37 (dd, *J* = 12.3, 7.2 Hz, 1H), 4.25–4.33 (m, 1H), 6.78 (d, *J* = 3.7 Hz, 1H), 7.03 (d, *J* = 7.2 Hz, 2H), 7.30 (t, *J* = 7.7 Hz, 2H), 7.39–7.46 (m, 2H), 7.62 (d, *J* = 3.4 Hz, 1H), 7.66–7.74 (m, 2H), 7.95 (d, *J* = 7.2 Hz, 1H). ^{13}C NMR (126 MHz, CD_3OD) δ ppm 29.5, 44.3, 48.9, 49.4, 110.7, 122.9 (q, *J* = 272.2 Hz), 124.2, 124.4, 127.4, 128.9, 129.3, 130.7, 130.9, 131.0, 131.1, 131.3, 131.8, 135.5, 139.1, 165.04. Monoisotopic Mass for free base $C_{22}H_{20}F_3N_3O_3S$: 463.12, $[M+H]^+$ 464.1.

(*S*)-2-Phenyl-1-[(3-trifluoromethylphenyl)sulfonyl]-*N*-(pyrrolidin-3-yl)-1*H*-pyrrole-3-carboxamide (14)

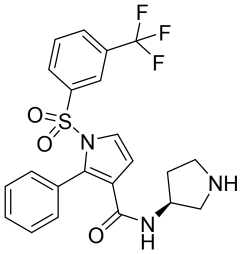

Figure S29. Chemical structure of compound **14**

Boc-derivative: colorless oil, 0.10 g (yield 79%) after chromatographic purification over silica gel with EtOAc/Hex (6/4, v/v); UPLC/MS purity 98%, t_R = 8.02, $C_{27}H_{28}F_3N_3O_5S$, MW 563.59, Monoisotopic Mass 563.17, $[M+H]^+$ 564.2.

Hydrochloride: white solid, 0.08 g (yield 90%); UPLC/MS purity 100%, t_R = 5.08, $C_{22}H_{21}ClF_3N_3O_3S$, MW 499.93. ^{1}H NMR (500 MHz, CD_3OD) δ ppm 1.72–1.81 (m, 1H), 2.11–2.20 (m, 1H), 3.02 (dd, *J* = 12.0, 4.9 Hz, 1H), 3.17–3.27 (m, 2H), 3.37 (dd, *J* = 12.2, 7.2 Hz, 1H), 4.25–4.33 (m, 1H), 6.78 (d, *J* = 3.7 Hz, 1H), 7.03 (d, *J* = 7.2 Hz, 2H), 7.30 (t, *J* = 7.7 Hz, 2H), 7.39–7.47 (m, 2H), 7.62 (d, *J* = 3.4 Hz, 1H), 7.66–7.77 (m, 2H), 7.95 (d, *J* = 7.2 Hz, 1H). ^{13}C NMR (126 MHz, CD_3OD) δ ppm 29.5, 44.3, 48.9, 49.5, 110.8, 122.9 (q, *J* = 272.1 Hz), 124.2, 124.4, 127.4, 128.9, 129.3, 130.7, 130.9, 131.0, 131.2, 131.3, 131.8, 135.5, 139.1, 165.04. Monoisotopic Mass for free base $C_{22}H_{20}F_3N_3O_3S$: 463.12, $[M+H]^+$ 464.1. Monoisotopic Mass for free base $C_{22}H_{20}F_3N_3O_3S$: 463.12, $[M+H]^+$ 464.1.

(*R*)-2-Phenyl-1-[(3-trifluoromethoxyphenyl)sulfonyl]-*N*-(pyrrolidin-3-yl)-1*H*-pyrrole-3-carboxamide (15)

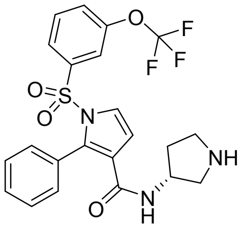

Figure S30. Chemical structure of compound **15**

Boc-derivative: colorless oil, 0.13 g (yield 64%) after chromatographic purification over silica gel with CH_2Cl_2/MeOH (9/0.2, v/v); UPLC/MS purity 100%, t_R = 8.13, $C_{27}H_{28}F_3N_3O_6S$, MW 579.59, Monoisotopic Mass 579.17, $[M+H]^+$ 580.2.

Hydrochloride: white solid, 0.07 g (yield 63%); UPLC/MS purity 100%, t_R = 5.16, $C_{22}H_{21}ClF_3N_3O_4S$, MW 515.93. ^{1}H NMR (500 MHz, CD_3OD) δ ppm 1.73–1.81 (m, 1H), 2.12–2.20 (m, 1H), 3.03 (dd, *J* = 12.2, 5.1 Hz, 1H), 3.18–3.28 (m, 2H), 3.37 (dd, *J* = 12.3, 7.2 Hz, 1H), 4.26–4.33 (m, 1H), 6.78 (d, *J* = 3.7 Hz, 1H), 7.02–7.06 (m, 2H), 7.08–7.12 (m, 1H), 7.30 (t, *J* = 7.9 Hz, 2H), 7.37–7.45 (m, 2H), 7.54–7.58 (m, 2H), 7.61 (d, *J* = 3.7 Hz, 1H). ^{13}C NMR (126 MHz, CD_3OD) δ ppm 29.5, 44.3, 48.9, 49.5, 110.7, 119.7, 120.3, 122.7, 123.0, 126.1, 126.7, 127.4, 129.0, 129.2, 131.5, 131.7, 135.6, 139.9, 148.9, 165.1. Monoisotopic Mass for free base $C_{22}H_{20}F_3N_3O_4S$ 479.11, $[M+H]^+$ 480.1.

(*S*)-2-Phenyl-1-[(3-trifluoromethoxyphenyl)sulfonyl]-*N*-(pyrrolidin-3-yl)-1*H*-pyrrole-3-carboxamide (16)

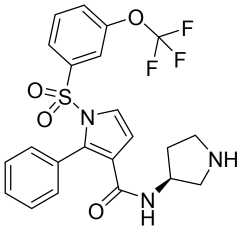

Figure S31. Chemical structure of compound **16**

Boc-derivative: colorless oil, 0.11 g (yield 67%) after chromatographic purification over silica gel with EtOAc/Hex (6/4, v/v); UPLC/MS purity 100%, t_R = 8.14, $C_{27}H_{28}F_3N_3O_6S$, MW 579.59, Monoisotopic Mass 579.17, $[M+H]^+$ 580.2.

Hydrochloride: white solid, 0.08 g (yield 82%); UPLC/MS purity 100%, t_R = 5.15, $C_{22}H_{21}ClF_3N_3O_4S$, MW 515.93. ^{1}H NMR (500 MHz, CD_3OD) δ ppm 1.71–1.80 (m, 1H), 2.10–2.19 (m, 1H), 3.01 (dd, *J* = 12.2, 5.0 Hz, 1H), 3.17–3.27 (m, 2H), 3.36 (dd, *J* = 12.2, 7.3 Hz, 1H), 4.24–4.30 (m, 1H), 6.76 (d, *J* = 3.4 Hz, 1H), 7.02–7.06 (m, 2H), 7.09–7.12 (m, 1H), 7.30 (t, *J* = 7.9 Hz, 2H), 7.37–7.45 (m, 2H), 7.53–7.57 (m, 2H), 7.59 (d, *J* = 3.7 Hz, 1H). ^{13}C NMR (126 MHz, CD_3OD) δ ppm 29.5, 44.3, 48.9, 49.5, 110.7, 119.8, 120.3, 122.7, 123.0, 126.0, 126.7, 127.4, 129.0, 129.2, 131.5, 131.7, 135.6, 139.9, 148.9, 165.1. Monoisotopic Mass for free base $C_{22}H_{20}F_3N_3O_4S$: 479.11, $[M+H]^+$ 480.1.

(*R*)-2-Phenyl-1-(3-chlorophenylsulfonyl)-*N*-(pyrrolidin-3-yl)-1*H*-pyrrole-3-carboxamide (17)

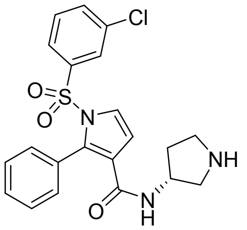

Figure S32. Chemical structure of compound **17**

Boc derivative: colorless oil, 0.18 g (yield 73%) after chromatographic purification over silica gel with EtOAc/Hex (6/4, v/v); UPLC/MS purity 97%, t_R = 7.75, $C_{26}H_{28}ClN_3O_5S$, MW 530.04, Monoisotopic Mass 529.14, $[M+H]^+$ 530.2.

Hydrochloride: white solid, 0.14 g (yield 88%); UPLC/MS purity 100%, t_R = 4.75, $C_{21}H_{21}Cl_2N_3O_3S$, MW 466.38. ^{1}H NMR (300 MHz, CD_3OD) δ ppm 1.72–1.80 (m, 1H), 2.10–2.22 (m, 1H), 3.02 (dd, *J* = 12.3, 5.1 Hz, 1H), 3.18–3.30 (m, 2H), 3.38 (dd, *J* = 12.3, 7.1 Hz, 1H), 4.28 (quint, *J* = 7.1 Hz, 1H), 6.73 (d, *J* = 3.5 Hz, 1H), 7.02–7.10 (m, 2H), 7.16 (t, *J* = 2.0 Hz, 1H), 7.30–7.38 (m, 3H), 7.44 (t, *J* = 8.0 Hz, 1H), 7.44–7.50 (m, 1H), 7.60 (d, *J* = 3.5 Hz, 1H), 7.65 (dd, *J* = 8.0, 2.0 Hz, 1H). ^{13}C NMR (CD_3OD, 75 MHz) δ ppm 30.7, 45.6, 50.2, 50.8, 111.7, 123.8, 124.2, 126.9, 128.6, 128.7, 130.3, 130.6, 132.2, 133.2, 135.7, 136.2, 136.7, 140.8, 166.5. Monoisotopic Mass for free base $C_{21}H_{20}ClN_3O_3S$: 429.09, $[M+H]^+$ 430.1.

(*S*)-2-Phenyl-1-[(thien-2-yl)sulfonyl]-*N*-(pyrrolidin-3-yl)-1*H*-pyrrole-3-carboxamide (19)

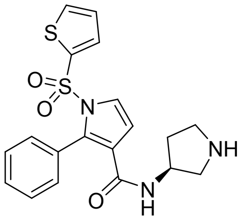

Figure S33. Chemical structure of compound **19**

Boc-derivative: colorless oil, 0.12 g (yield 85%) after chromatographic purification over silica gel with EtOAc/Hex (8/2, v/v); UPLC/MS purity 97%, t_R = 7.12, $C_{24}H_{27}N_3O_5S_2$, MW 501.62, Monoisotopic Mass 501.14, $[M+H]^+$ 502.1.

Hydrochloride: white solid, 0.07 g (yield 67%), UPLC/MS purity 100%, t_R = 4.08, $C_{19}H_{20}ClN_3O_3S_2$, MW 437.96. ^{1}H NMR (500 MHz, CD_3OD) δ ppm 1.68–1.79 (m, 1H), 2.08–2.19 (m, 1H), 3.01 (dd, *J* = 11.6, 3.6 Hz, 1H), 3.15–3.26 (m, 1H), 3.31–3.40 (m, 1H), 4.24–4.34 (m, 1H), 6.73 (d, *J* = 3.4 Hz, 1H), 6.98 (t, *J* = 4.4 Hz, 1H), 7.07 (dd, *J* = 3.7, 0.9 Hz, 1H), 7.12 (d, *J* = 7.5 Hz, 2H), 7.33 (t, *J* = 7.6 Hz, 2H), 7.43 (t, *J* = 7.2 Hz, 1H), 7.48 (d, *J* = 3.4 Hz, 1H), 7.86 (dd, *J* = 4.8, 0.9 Hz, 1H). ^{13}C NMR (126 MHz, CD_3OD) δ ppm 29.5, 44.3, 48.8, 49.5, 110.6, 122.2, 122.7, 127.4, 127.5, 129.1, 129.2, 131.7, 135.2, 135.6, 135.9, 137.7, 165.2. Monoisotopic Mass for free base $C_{19}H_{19}N_3O_3S_2$: 401.09, $[M+H]^+$ 402.1.

(*S*)-2-Phenyl-1-[(1-methyl-1*H*-pyrazol-4-yl)sulfonyl]-*N*-(pyrrolidin-3-yl)-1*H*-pyrrole-3-carboxamide (20)

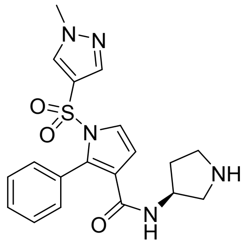

Figure S34. Chemical structure of compound **20**

Boc-derivative: colorless oil, 0.09 g (yield 64%) after chromatographic purification over silica gel with EtOAc/Hex (8/2, v/v); UPLC/MS purity 97%, t_R = 6.24, $C_{24}H_{29}N_5O_5S$, MW 499.59, Monoisotopic Mass 499.19, $[M+H]^+$ 500.2.

Hydrochloride: white solid, 0.06 g (yield 76%), UPLC/MS purity 100%, t_R = 3.27, $C_{19}H_{22}ClN_5O_3S$, MW 435.93. ^{1}H NMR (500 MHz, CD_3OD) δ ppm 1.73–1.83 (m, 1H), 2.12–2.22 (m, 1H), 3.05 (dd, *J* = 12.0, 4.6 Hz, 1H), 3.19–3.29 (m, 2H), 3.39 (dd, *J* = 12.2, 7.0 Hz, 1H), 3.77 (s, 3H), 4.28–4.36 (m, 1H), 6.71 (d, *J* = 3.4 Hz, 1H), 7.17 (d, *J* = 7.2 Hz, 2H), 7.26 (s, 1H), 7.39 (t, *J* = 7.7 Hz, 2H), 7.46–7.49 (m, 2H), 7.50 (s, 1H). ^{13}C NMR (126 MHz, CD_3OD) δ ppm 29.5, 38.3, 44.3, 48.8, 49.5, 109.7, 119.7, 121.7, 122.2, 127.4, 129.0, 129.7, 131.8, 134.1, 135.1, 138.4, 165.3. Monoisotopic Mass for free base $C_{19}H_{21}N_5O_3S$: 399.14, $[M+H]^+$ 400.1.

(*R*)-2-(3-Fluorophenyl)-1-[(3-chlorophenyl)sulfonyl)-*N*-(pyrrolidin-3-yl)-1*H*-pyrrole-3-carboxamide (21)

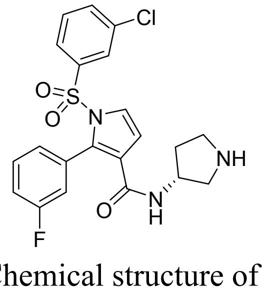

Figure S35. Chemical structure of compound **21**

Boc-derivative: colorless oil, 0.18 g (yield 68%) after chromatographic purification over silica gel with CH_2Cl_2/MeOH (9/0.3, v/v); UPLC/MS purity 100%, t_R = 7.86, $C_{26}H_{27}ClFN_3O_5S$, MW 548.03, Monoisotopic Mass 547.13, $[M+H]^+$ 548.2.

Hydrochloride: white solid, 0.11 g (yield 69%); UPLC/MS purity 99%, t_R = 4.93, $C_{21}H_{20}Cl_2FN_3O_3S$, MW 484.37. ^{1}H NMR (400 MHz, CD_3OD) δ ppm 1.80–1.98 (m, 1H), 2.16–2.30 (m, 1H), 3.08 (dd, *J* = 12.2 Hz, 5.1 Hz, 1H), 3.20–3.28 (m, 1H), 3.33–3.38 (m, 1H), 3.41 (dd, *J* = 12.1 Hz, 7.2 Hz, 1H), 4.30 (quint, *J* = 7.1 Hz, 1H), 6.75 (d, *J* = 3.5 Hz, 1H), 6.78 (dt, *J* = 9.0 Hz, 2.3 Hz, 1H), 6.84 (d, *J* = 7.6 Hz, 1H), 7.21 (td, *J* = 9.4 Hz, 2.0 Hz, 1H), 7.25 (t, *J* = 2.0 Hz, 1H), 7.29–7.41 (m, 2H), 7.47 (t, *J* = 8.0 Hz, 1H), 7.62 (d, *J* = 3.5 Hz, 1H), 7.68 (dd, *J* = 8.0 Hz, 1.0 Hz, 1H). ^{13}C NMR (100 MHz, CD_3OD) δ ppm 30.7, 45.7, 50.3, 50.8, 111.6, 117.2 (d, *J* = 21.3 Hz), 120.1 (d, *J* = 22.7 Hz), 124.1, 126.9, 127.2, 128.6, 129.1, 130.2

(d, $J = 8.8$ Hz), 132.3, 135.3, 135.4, 135.8, 136.3, 140.8, 163.1 (d, $J = 243.9$ Hz), 166.2. Monoisotopic Mass for free base $C_{21}H_{19}ClFN_3O_3S$ 447.08, $[M+H]^+$ 448.1.

(*R*)-2-(3-Fluorophenyl)-1-[(4-fluorophenyl)sulfonyl]-*N*-(pyrrolidin-3-yl)-1*H*-pyrrole-3-carboxamide (23)

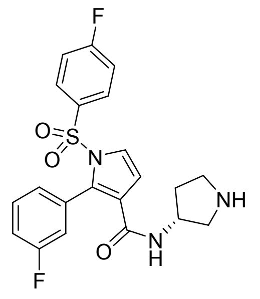

Figure S36. Chemical structure of compound **23**

Boc-derivative: colorless oil, 0.09 g (yield 63%) after chromatographic purification over silica gel with CH_2Cl_2/MeOH (9/0.2, v/v); UPLC/MS purity 100%, t_R = 7.58, $C_{26}H_{27}F_2N_3O_5S$, MW 531.57, Monoisotopic Mass 531.16, $[M+H]^+$ 532.2.

Hydrochloride: white solid, 0.07 g (yield 78%), UPLC/MS purity 100%, $t_R = 4.68$, $C_{21}H_{20}ClF_2N_3O_3S$, MW 467.92. ^1H NMR (400 MHz, CD_3OD) δ ppm 1.80–1.96 (m, 1H), 2.16–2.28 (m, 1H), 3.10 (dd, J = 12.2 Hz,4.9 Hz, 1H), 3.22–3.28 (m, 1H), 3.33–3.39 (m, 1H), 3.41 (dd, J = 12.2 Hz, 7.1 Hz, 1H), 4.26–4.40 (m, 1H), 6.75(d, J = 3.5 Hz, 1H), 6.80 (dt, J = 9.4 Hz, 2.1 Hz, 1H), 6.86 (d, J = 7.6 Hz, 1H), 7.16–7.24 (m, 3H), 7.28–7.38 (m, 1H), 7.41–7.49 (m, 2H), 7.60 (d, J = 3.5 Hz, 1H). ^{13}C NMR (100 MHz, CD_3OD) δ ppm 30.8, 45.6, 50.3, 50.7, 111.4, 117.1 (d, J = 21.3 Hz), 117.7 (d, J = 23.5 Hz), 120.0 (d, J = 22.7 Hz), 124.1, 124.2, 129.0 (d, J = 2.9 Hz), 130.2 (d, J = 8.1 Hz), 131.9 (d, J = 10.3 Hz), 132.7 (d, J = 8.8 Hz), 135.3, 135.4, 163.1 (d, J = 245.0 Hz), 166.2, 167.5 (d, J = 253.1 Hz). Monoisotopic Mass for free base $C_{21}H_{19}F_2N_3O_3S$: 431.11, $[M+H]^+$ 432.2.

(*R*)-2-(4-Fluorophenyl)-1-(phenylsulfonyl)-*N*-(pyrrolidin-3-yl)-1*H*-pyrrole-3-carboxamide (24)

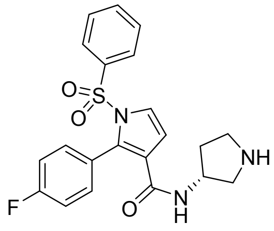

Figure S37. Chemical structure of compound **24**

Boc-derivative: colorless oil, 0.09 mg (yield 65%) after chromatographic purification over silica gel with CH_2Cl_2/MeOH (9/0.2, v/v); UPLC/MS purity 99%, t_R = 7.45, $C_{26}H_{28}FN_3O_5S$, MW 513.58, Monoisotopic Mass 513.17, $[M+H]^+$ 514.2.

Hydrochloride: white solid, 0.06 g (yield 76%), UPLC/MS purity 100%, $t_R = 4.48$, $C_{23}H_{21}F_4N_3O_5S$, MW 527.49. ^1H NMR (300 MHz, CD_3OD) δ ppm 1.81–1.89 (m, 1H), 2.16–

2.18 (m, 1H), 3.09 (dd, J = 12.1, 5.1 Hz, 1H), 3.24–3.28 (m, 1H), 3.32–3.36 (m, 1H), 3.40 (dd, J = 12.1, 7.2 Hz, 1H), 4.30 (quint, J = 7.0 Hz, 1H), 6.72 (d, J = 3.5 Hz, 1H), 6.96–7.12 (m, 4H), 7.33–7.41 (m, 2H), 7.42–7.48 (m, 2H), 7.60 (d, J = 3.5 Hz, 1H), 7.65 (tdd, J = 7.4, 1.4, 1.2 Hz, 1H). ^{13}C NMR (75 MHz, CD_3OD) δ ppm 30.7, 45.7, 50.2, 50.8, 111.3, 115.3 (d, J = 21.6 Hz), 123.9, 124.1, 126.7, 128.5, 130.5, 135.2 (d, J = 8.8 Hz), 135.7, 135.9, 139.4, 164.7 (d, J = 247.7 Hz), 166.5. Monoisotopic Mass for free base $C_{21}H_{20}FN_3O_3S$: 413.12, $[M+H]^+$ 414.3.

(*R*)-2-(4-Fluorophenyl)-1-[(3-methoxyphenyl)sulfonyl]-*N*-(pyrrolidin-3-yl)-1*H*-pyrrole-3-carboxamide (25)

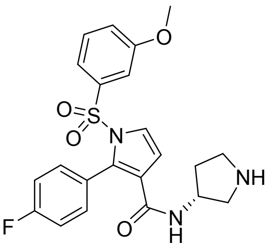

Figure S38. Chemical structure of compound **25**

Boc-derivative: colorless oil, 0.14 g (yield 84%) after chromatographic purification over silica gel with CH_2Cl_2/MeOH (9/0.2, v/v); UPLC/MS purity 100%, t_R = 7.5, $C_{27}H_{30}FN_3O_6S$, MW 543.61, Monoisotopic Mass 543.18, $[M+H]^+$ 544.2.

Hydrochloride: white solid, 0.10 g (yield 81%), UPLC/MS purity 100%, t_R = 4.65, $C_{22}H_{23}ClFN_3O_4S$, MW 479.95. ^{1}H NMR (500 MHz, CD_3OD) δ ppm 1.83–1.92 (m, 1H), 2.15–2.25 (m, 1H), 3.09 (dd, J = 12.0, 4.6 Hz, 1H), 3.20–3.28 (m, 1H), 3.32–3.43 (m, 2H), 3.72 (s, 3H), 4.27–4.36 (m, 1H), 6.74–6.78 (m, 2H), 6.98 (dd, J = 7.7, 1.2 Hz, 1H), 7.00–7.08 (m, 4H), 7.18 (dd, J = 8.3, 2.0 Hz, 1H), 7.36 (t, J = 8.0 Hz, 1H), 7.57 (d, J = 3.4 Hz, 1H). ^{13}C NMR (126 MHz, CD_3OD) δ ppm 29.5, 44.4, 48.9, 49.5, 54.9, 110.1, 112.0, 114.0 (d, J = 21.1 Hz), 119.1, 120.3, 122.6, 122.7, 125.4 (d, J = 3.6 Hz), 130.4, 134.0 (d, J = 8.5 Hz), 134.7, 139.0, 159.9, 164.3 (d, J = 249.3 Hz), 165.1. Monoisotopic Mass for free base $C_{22}H_{22}FN_3O_4S$: 443.13, $[M+H]^+$ 444.15.

(*R*)-2-(4-Fluorophenyl)-1-[(3-fluorophenyl)sulfonyl]-*N*-(pyrrolidin-3-yl)-1*H*-pyrrole-3-carboxamide (26)

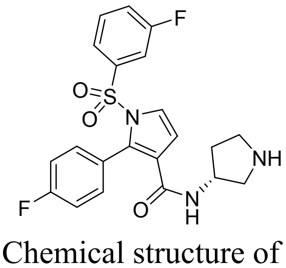

Figure S39. Chemical structure of compound **26**

Boc-derivative: colorless oil, 0.15 g (yield 92%) after chromatographic purification over silica gel with CH_2Cl_2/MeOH (9/0.2, v/v); UPLC/MS purity 100%, t_R = 7.50, $C_{26}H_{27}F_2N_3O_5S$, MW 531.57, Monoisotopic Mass 531.16, $[M+H]^+$ 532.2.

Hydrochloride: white solid, 0.11 g (yield 83%), UPLC/MS purity 100%, $t_R = 4.61$, $C_{21}H_{20}ClF_2N_3O_3S$, MW 467.92. ^{1}H NMR (300 MHz, CD_3OD) δ ppm 1.81–1.91 (m, 1H), 2.16–2.26 (m, 1H), 3.07 (dd, *J* = 12.2, 5.1 Hz, 1H), 3.21–3.27 (m, 1H), 3.33–3.37 (m, 1H), 3.40 (dd, *J* = 12.2, 7.2 Hz, 1H), 4.29 (quint, *J* = 7.2 Hz, 1H), 6.74 (d, *J* = 3.5 Hz, 1H), 6.98–7.14 (m, 5H), 7.24 (ddd, *J* = 7.7, 1.7, 1.1 Hz, 1H), 7.43 (td, *J* = 8.1, 1.0 Hz, 1H), 7.46–7.55 (m, 1H), 7.61 (d, *J* = 3.5 Hz, 1H). ^{13}C NMR (75 MHz, CD_3OD) δ ppm 30.7, 45.7, 50.3, 50.8, 111.6, 115.5 (d, *J* = 22.4 Hz), 115.7 (d, *J* = 26.4 Hz), 122.8, 123.0, 124.2 (d, *J* = 24.8 Hz), 124.6, 126.5 (d, *J* = 3.2 Hz), 132.8 (d, *J* = 8.0 Hz), 135.3 (d, *J* = 8.8 Hz), 135.9, 141.1 (d, *J* = 7.2 Hz), 164.8 (d, *J* = 248.5 Hz, 2C), 166.4. Monoisotopic Mass for free base $C_{21}H_{19}F_2N_3O_3S$: 431.11, $[M+H]^+$ 432.1.

(*R*)-2-(4-Fluorophenyl)-1-[(4-fluorophenyl)sulfonyl]-*N*-(pyrrolidin-3-yl)-1*H*-pyrrole-3-carboxamide (29)

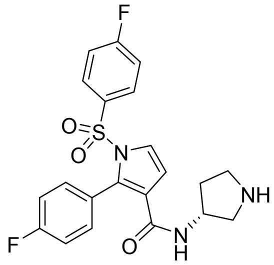

Figure S40. Chemical structure of compound **29**

Boc-derivative: colorless oil, 0.10 g (yield 70%) after chromatographic purification over silica gel with CH_2Cl_2/MeOH (9/0.2, v/v); UPLC/MS purity 99%, $t_R = 7.53$, $C_{26}H_{27}F_2N_3O_5S$, MW 531.57, Monoisotopic Mass 531.16, $[M+H]^+$ 532.2.

Hydrochloride: white solid, 0.08 g (yield 78%), UPLC/MS purity 100%, $t_R = 4.64$, $C_{21}H_{20}ClF_2N_3O_3S$, MW 467.92. ^{1}H NMR (400 MHz, CD_3OD) δ ppm 1.50–1.58 (m, 1H), 2.15–2.27 (m, 1H), 3.06 (dd, *J* = 12.2 Hz, 5.1 Hz, 1H), 3.22–3.28 (m, 1H), 3.32–3.36 (m, 1H), 3.41 (dd, *J* = 12.2 Hz, 7.1 Hz, 1H), 4.29 (quint, *J* = 6.7 Hz, 1H), 6.72 (d, *J* = 3.5 Hz, 1H), 7.01–7.11 (m, 4H), 7.20 (t, *J* = 8.6 Hz, 2H), 7.37–7.50 (m, 2H), 7.59 (d, *J* = 3.5 Hz, 1H). ^{13}C NMR (100 MHz, CD_3OD) δ ppm 30.8, 45.7, 50.3, 50.8, 111.5, 115.5 (d, *J* = 22.0 Hz), 117.7 (d, *J* = 23.5 Hz), 123.9, 124.2, 132.8 (d, *J* = 10.3 Hz), 135.2 (d, *J* = 8.8 Hz), 135.5, 135.8, 164.1 (d, *J* = 298.7 Hz), 164.8 (d, *J* = 269.7 Hz), 166.4. Monoisotopic Mass for free base $C_{21}H_{19}F_2N_3O_3S$: 431.11, $[M+H]^+$ 432.1.

(*S*)-2-(4-Fluorophenyl)-1-[(4-fluorophenyl)sulfonyl]-*N*-(pyrrolidin-3-yl)-1*H*-pyrrole-3-carboxamide (30)

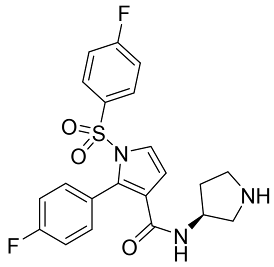

Figure S41. Chemical structure of compound **30**

Boc-derivative: colorless oil, 0.08 g (yield 56%) after chromatographic purification over silica gel with CH_2Cl_2/MeOH (9/0.2, v/v); UPLC/MS purity 100%, t_R = 7.54, $C_{26}H_{27}F_2N_3O_5S$, MW 531.57, Monoisotopic Mass 531.16, $[M+H]^+$ 532.2.

Hydrochloride: white solid, 0.09 g (yield 85%), UPLC/MS purity 100%, t_R = 4.65, $C_{21}H_{20}ClF_2N_3O_3S$, MW 467.92. ^{1}H NMR (400 MHz, CD_3OD) δ ppm 1.48–1.60 (m, 1H), 2.16–2.26 (m, 1H), 3.06 (dd, *J* = 12.2 Hz, 5.1 Hz, 1H), 3.21–3.29 (m, 1H), 3.30–3.34 (m, 1H), 3.41 (dd, *J* = 12.2 Hz, 7.1 Hz, 1H), 4.29 (quint, *J* = 6.7 Hz, 1H), 6.72 (d, *J* = 3.5 Hz, 1H), 7.01–7.11 (m, 4H), 7.20 (t, *J* = 8.6 Hz, 2H), 7.39–7.47 (m, 2H), 7.59 (d, *J* = 3.5 Hz, 1H). ^{13}C NMR (100 MHz, CD_3OD) δ ppm 30.8, 45.7, 50.3, 50.8, 111.5, 115.5 (d, *J* = 22.0 Hz), 117.7 (d, *J* = 23.5 Hz), 123.9, 124.2, 132.8 (d, *J* = 10.3 Hz), 135.2 (d, *J* = 8.8 Hz), 135.5, 135.8, 164.1 (d, *J* = 298.7 Hz), 164.8 (d, *J* = 269.7 Hz), 166.4. Monoisotopic Mass for free base $C_{21}H_{19}F_2N_3O_3S$: 431.11, $[M+H]^+$ 432.1.

(*S*)-2-(4-Fluorophenyl)-1-[(naphth-1-yl)sulfonyl]-*N*-(pyrrolidin-3-yl)-1*H*-pyrrole-3-carboxamide (31)

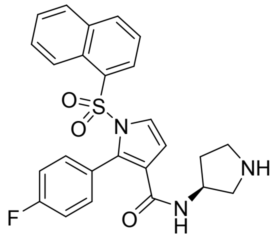

Figure S42. Chemical structure of compound **31**

Boc-derivative: colorless oil, 0.13 g (yield 86%) after chromatographic purification over silica gel with EtOAc/Hex (6/4, v/v); UPLC/MS purity 100%, t_R = 7.93, $C_{30}H_{30}FN_3O_5S$, Molecular Weight 563.64, Monoisotopic Mass 563.19, $[M+H]^+$ 564.2.

Hydrochloride: white solid, 0.08 g (yield 69%); UPLC/MS purity 100%, t_R = 5.06, $C_{25}H_{23}ClFN_3O_3S$, Molecular Weight 499.99. ^{1}H NMR (500 MHz, CD_3OD) δ ppm 1.77–1.86 (m, 1H), 2.09–2.19 (m, 1H), 3.02 (dd, *J* = 12.2, 4.7 Hz, 1H), 3.14–3.24 (m, 1H), 3.30–3.37 (m, 2H), 4.20–4.29 (m, 1H), 6.62–6.69 (m, 2H), 6.76–6.84 (m, 3H), 7.23 (t, *J* = 7.9 Hz, 1H), 7.43 (dd, *J* = 7.5, 1.2 Hz, 1H), 7.55–7.63 (m, 2H), 7.84 (d, *J* = 3.4 Hz, 1H), 7.97–8.01 (m, 1H), 8.13 (d, *J* = 8.3 Hz, 1H), 8.18–8.22 (m, 1H).^{13}C NMR (126 MHz, CD_3OD) δ ppm 29.4, 44.3, 48.8, 49.5, 109.3, 114.0 (d, *J* = 22.3 Hz), 121.9, 122.1, 122.5, 123.8, 125.1 (d, *J* = 3.6 Hz), 127.1, 127.4, 128.8, 129.2, 131.4, 132.1, 133.5 (d, *J* = 9.1 Hz), 134.0, 134.4, 136.1,

163.1 (d, $J = 242.0$ Hz), 164.93. Monoisotopic Mass for free base $C_{25}H_{22}FN_3O_3S$: 463.14, $[M+H]^+$ 464.1.

(*S*)-2-(4-Fluorophenyl)-1-[(quinol-8-yl)sulfonyl]-*N*-(pyrrolidin-3-yl)-1*H*-pyrrole-3-carboxamide (32)

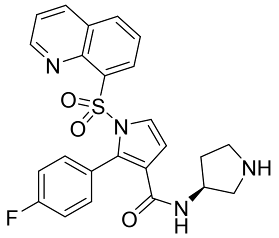

Figure S43. Chemical structure of compound **32**

Boc-derivative: colorless oil, 0.14 g (yield 92%) after chromatographic purification over silica gel with EtOAc/Hex (8/2, v/v); UPLC/MS purity 100%, $t_R = 7.05$, $C_{29}H_{29}FN_4O_5S$, MW 564.63, Monoisotopic Mass 564.18, $[M+H]^+$ 565.2.

Hydrochloride: white solid, 0.05 g (yield 74%); UPLC/MS purity 98%, $t_R = 4.25$, $C_{24}H_{22}ClFN_4O_3S$, MW 500.97. ^1H NMR (500 MHz, CD_3OD) δ ppm 1.78–1.87 (m, 1H), 2.12–2.21 (m, 1H), 2.99–3.06 (m, 1H), 3.17–3.25 (m, 1H), 3.31–3.39 (m, 1H), 4.20–4.27 (m, 1H), 6.62–6.69 (m, 3 H), 6.81 (t, $J = 8.8$ Hz, 2H), 7.44 (dd, $J = 8.0$, 7.5 Hz, 1H), 7.59 (dd, $J = 8.3$, 4.3 Hz, 1H), 7.70 (dd, $J = 7.6$, 1.3 Hz, 1H), 7.85 (d, $J = 3.7$ Hz, 1H), 8.22 (dd, $J = 8.3$, 1.4 Hz, 1H), 8.39 (dd, $J = 8.5$, 1.6 Hz, 1H), 8.86 (dd, $J = 4.3$, 1.7 Hz, 1H). ^{13}C NMR (126 MHz, CD_3OD) δ ppm 29.4, 44.4, 48.9, 49.5, 107.6, 113.9 (d, $J = 21.7$ Hz), 121.4, 122.6, 124.4, 124.9, 125.6 (d, $J = 3.6$ Hz), 128.9, 133.1, 133.5 (d, $J = 8.5$ Hz), 134.0, 134.1, 135.8, 136.6, 143.1, 151.7, 163.0 (d, $J = 250.5$ Hz), 165.3. Monoisotopic Mass for free base $C_{24}H_{21}FN_4O_3S$: 464.13, $[M+H]^+$ 465.1.

4. Spectra of selected final compound 27

4.1 HRMS spectra

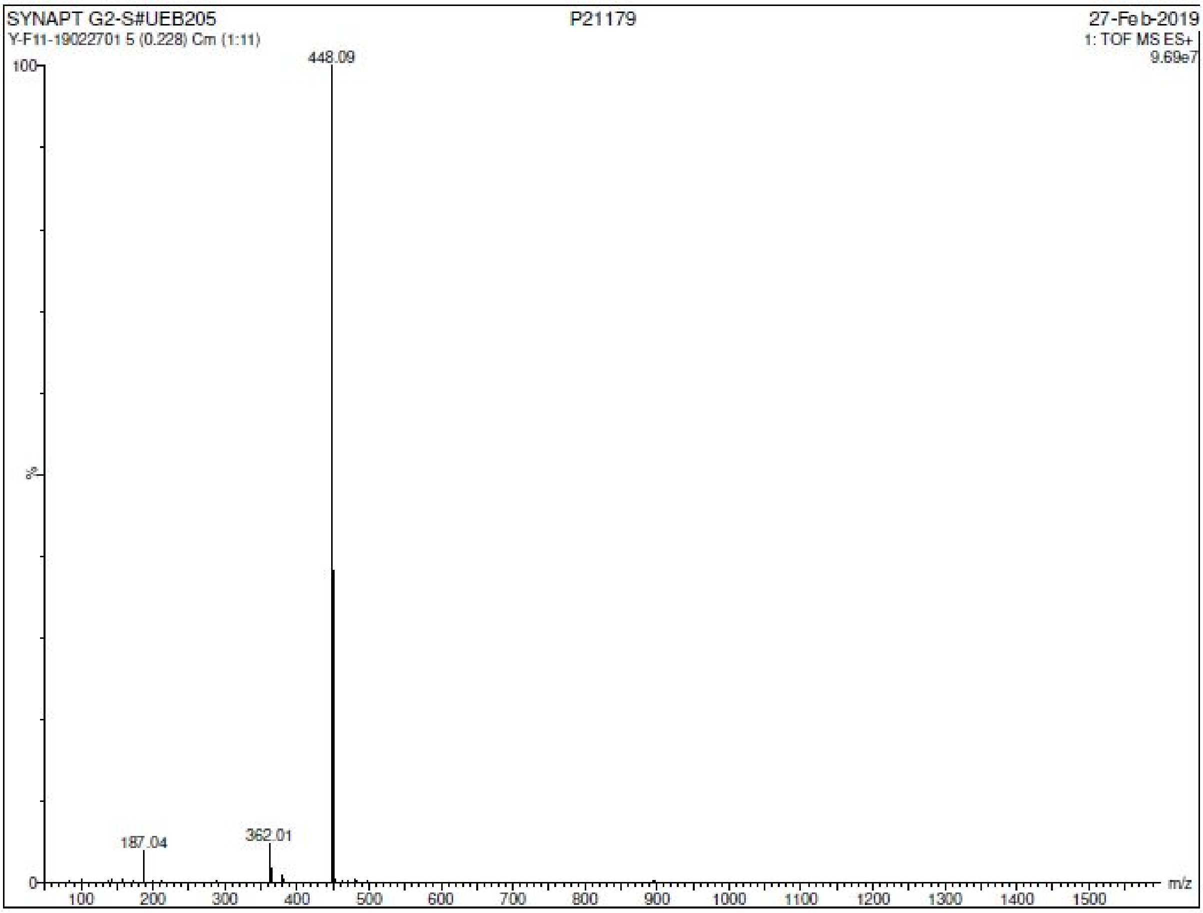

Single Mass Analysis
Tolerance = 3.0 mDa / DBE: min = -1.5, max = 50.0
Element prediction: Off
Number of isotope peaks used for i-FIT = 3

Monoisotopic Mass, Even Electron Ions
906 formula(e) evaluated with 7 results within limits (up to 50 best isotopic matches for each mass)
Elements Used:
C: 0-200 H: 0-200 N: 0-30 O: 0-30 S: 1-1 Cl: 1-1 F: 1-1

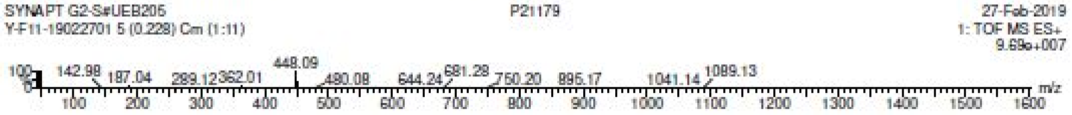

Mass	Calc. Mass	mDa	PPM	DBE	i-FIT	Norm	Conf(%)	Formula
Minimum:				-1.5				
Maximum:		3.0	2.0	50.0				
448.0896	448.0898	-0.2	-0.4	12.5	3012.3	0.037	96.37	C21 H20 N3 O3 S Cl F
	448.0871	2.5	5.6	13.5	3015.6	3.318	3.62	C17 H16 N9 O S Cl F
	448.0916	-2.0	-4.5	-0.5	3022.5	10.197	0.00	C9 H24 N5 O10 S Cl F
	448.0916	-2.0	-4.5	10.5	3024.6	12.324	0.00	C7 H12 N19 S Cl F
	448.0903	-0.7	-1.6	5.5	3025.3	12.980	0.00	C6 H16 N15 O4 S Cl F
	448.0890	0.6	1.3	0.5	3025.9	13.585	0.00	C5 H20 N11 O8 S Cl F
	448.0876	2.0	4.5	6.5	3029.0	16.663	0.00	C2 H12 N21 O2 S Cl F

Figure S44. HRMS spectra for **27**: (*R*)-2-(4-Fluorophenyl)-1-[(3-chlorophenyl)sulfonyl]-*N*-(pyrrolidin-3-yl)-1*H*-pyrrole-3-carboxamide.

4.2 ^{1}H NMR and ^{13}C NMR spectra

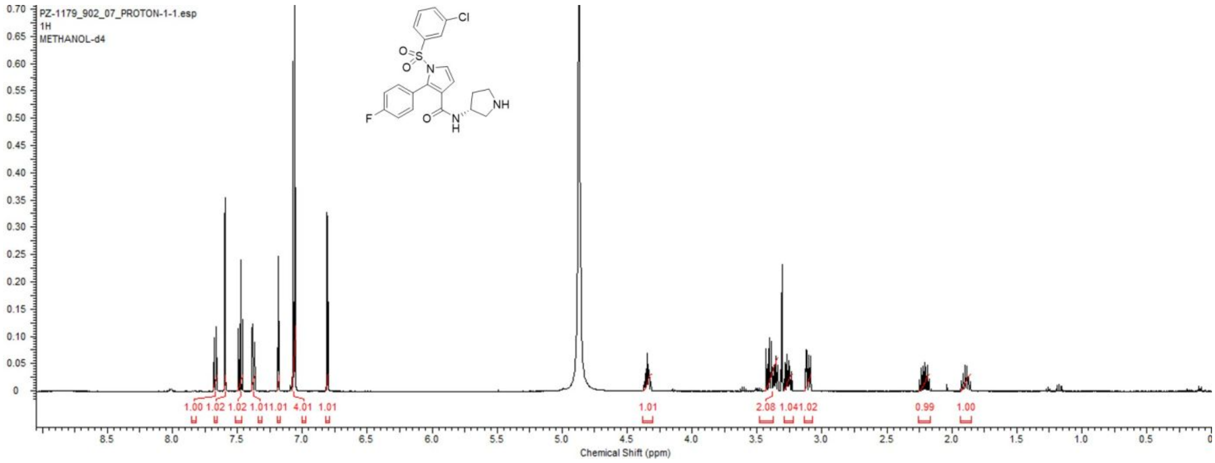

Figure S45. ^{1}H NMR spectra for **27**: (*R*)-2-(4-Fluorophenyl)-1-[(3-chlorophenyl)sulfonyl]-*N*-(pyrrolidin-3-yl)-1*H*-pyrrole-3-carboxamide.

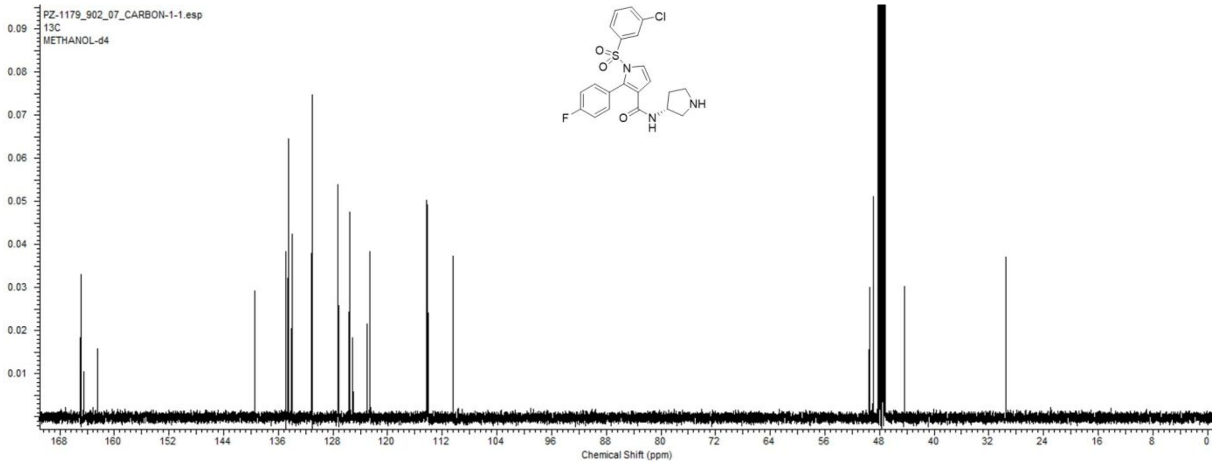

Figure S46. ^{13}C NMR spectra for **27**: (*R*)-2-(4-Fluorophenyl)-1-[(3-chlorophenyl)sulfonyl]-*N*-(pyrrolidin-3-yl)-1*H*-pyrrole-3-carboxamide.

5. **The composition of the buffers used in radioligand binding assay**

5.1 Buffer for 5-HT$_{1A}$R

50 mM Tris HCl, 0.1 mM EDTA, 4 mM $MgCl_2$, 10 µM pargyline and 0.1% ascorbate

5.2 Buffer for 5-HT$_{2A}$R

50 mM Tris HCl, 0.1 mM EDTA, 4 mM $MgCl_2$ and 0.1% ascorbate

5.3 Buffer for 5-HT$_6$R

50 mM Tris HCl, 0.5 mM EDTA and 4 mM $MgCl_2$

5.4 Buffer for 5-HT$_{7b}$R

50 mM Tris HCl, 4 mM $MgCl_2$, 10 µM pargyline and 0.1% ascorbate

5.5 Buffer for dopamine D$_{2L}$R

50 mM Tris HCl, 1 mM EDTA, 4 mM $MgCl_2$, 120 mM NaCl, 5 mM KCl, 1.5 mM $CaCl_2$ and 0.1% ascorbate

6. ***In vitro* safety space assessment for compound 27**

6.1 Evaluation of CYP450 inhibition

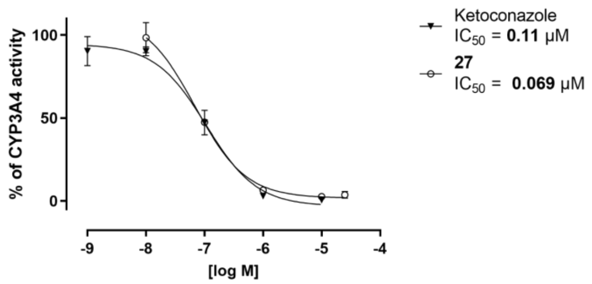

Figure S47. The effect of **27** and the reference inhibitor ketoconazole on CYP3A4 activity. IC_{50} values were calculated using GraphPad Prism 5 Software.

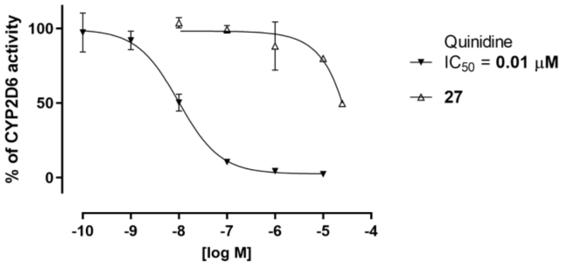

Figure S48. The effect of **27** and the reference inhibitor quinidine on CYP2D6 activity. IC_{50} value of quinidine was calculated using GraphPad Prism 5 Software. IC_{50} for **27** was not calculated.

6.2 Evaluation of mutagenic properties

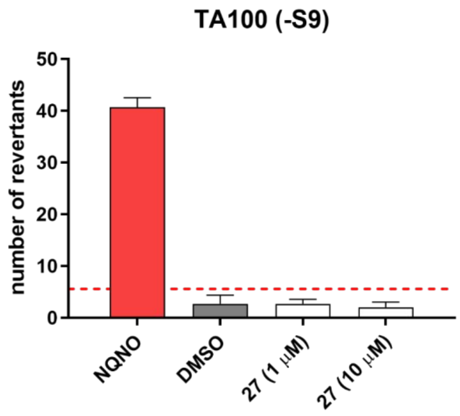

Figure S49. Ames test. The number of revertants of the *Salmonella typhimurium* TA100 strain (without S9 fraction) exposed to the reference mutagen nonyl-4-hydroxyquinoline-N-oxide (NQNO, 0.5 μM) and compound **27** in two concentrations of 1 and 10 μM. DMSO (1% in bacteria medium) was used as a negative control. The dashed line marks the doubled medium control baseline considered as the mutagen alert. The compounds were examined in triplicate.

7. References

(1) Grychowska, K.; Kubica, B.; Drop, M.; Colacino, E.; Bantreil, X.; Pawłowski, M.; Martinez, J.; Subra, G.; Zajdel, P.; Lamaty, F. Application of the Ring-Closing Metathesis to the Formation of 2-Aryl-1H-Pyrrole-3-Carboxylates as Building Blocks for Biologically Active Compounds. *Tetrahedron* **2016**, *72*, 7462–7469. https://doi.org/10.1016/j.tet.2016.09.059.

(2) Sun, W.; Ma, X.; Hong, L.; Wang, R. Asymmetric Organocatalytic Allylic Substitution of Morita–Baylis–Hillman Carbonates with Allylamines for the Synthesis of 2,5-Dihydropyrroles. *J. Org. Chem.* **2011**, *76*, 7826–7833. https://doi.org/10.1021/jo2011522.

(3) Drop, M.; Bantreil, X.; Grychowska, K.; Mahoro, G. U.; Colacino, E.; Pawłowski, M.; Martinez, J.; Subra, G.; Zajdel, P.; Lamaty, F. Continuous Flow Ring-Closing Metathesis, an Environmentally-Friendly Route to 2,5-Dihydro-1H-Pyrrole-3-Carboxylates. *Green Chem.* **2017**, *19*, 1647–1652. https://doi.org/10.1039/C7GC00235A.

(4) Declerck, V.; Ribière, P.; Martinez, J.; Lamaty, F. Sequential Aza-Baylis−Hillman/Ring Closing Metathesis/Aromatization as a Novel Route for the Synthesis of Substituted Pyrroles. *J. Org. Chem.* **2004**, *69*, 8372–8381. https://doi.org/10.1021/jo048519r.
